# A Novel TGF-β Risk Score Predicts the Clinical Outcomes and Tumour Microenvironment Phenotypes in Bladder Cancer

**DOI:** 10.3389/fimmu.2021.791924

**Published:** 2021-12-17

**Authors:** Zhi Liu, Tiezheng Qi, Xiaowen Li, Yiyan Yao, Belaydi Othmane, Jinbo Chen, Xiongbing Zu, Zhenyu Ou, Jiao Hu

**Affiliations:** ^1^ Departments of Urology, Xiangya Hospital, Central South University, Changsha, China; ^2^ Departments of Urology, The Second Affiliated Hospital, Guizhou Medical University, Kaili, China; ^3^ National Clinical Research Center for Geriatric Disorders, Xiangya Hospital, Changsha, China; ^4^ Xiangya School of Medicine, Central South University, Changsha, China

**Keywords:** bladder cancer, tumour microenvironment, TGF-β, immunotherapy, risk score, chemotherapy

## Abstract

**Background:**

The TGF-β pathway plays critical roles in numerous malignancies. Nevertheless, its potential role in prognosis prediction and regulating tumour microenvironment (TME) characteristics require further elucidation in bladder cancer (BLCA).

**Methods:**

TGF-β-related genes were comprehensively summarized from several databases. The TCGA-BLCA cohort (training cohort) was downloaded from the Cancer Genome Atlas, and the independent validation cohorts were gathered from Xiangya Hospital (Xinagya cohort) and Gene Expression Omnibus. Initially, we identified differentially expressed TGF-β genes (DEGs) between cancer and normal tissues. Subsequently, univariate Cox analysis was applied to identify prognostic DEGs, which were further used to develop the TGF-β risk score by performing LASSO and multivariate Cox analyses. Then, we studied the role of the TGF-β risk score in predicting prognosis and the TME phenotypes. In addition, the role of the TGF-β risk score in guiding precision treatments for BLCA has also been assessed.

**Results:**

We successfully constructed a TGF-β risk score with an independent prognostic prediction value. A high TGF-β risk score indicated an inflamed TME, which was supported by the positive relationships between the risk score, enrichment scores of anticancer immunity steps, and the infiltration levels of tumour-infiltrating immune cells. In addition, the risk score positively correlated with the expression of several immune checkpoints and the T cell inflamed score. Consistently, the risk score was positively related to the enrichment scores of most immunotherapy-positive pathways. In addition, the sensitivities of six common chemotherapeutic drugs were positively associated with the risk score. Furthermore, higher risk score indicated higher sensitivity to radiotherapy and EGFR-targeted therapy. On the contrary, patients with low-risk scores were more sensitive to targeted therapies, including the blockade of FGFR3 and WNT-β-catenin networks.

**Conclusions:**

We first constructed and validated a TGF-β signature that could predict the prognosis and TME phenotypes for BLCA. More importantly, the TGF-β risk score could aid in individual precision treatment for BLCA.

## Introduction

Bladder cancer (BLCA) is the second frequently diagnosed urinary cancers with high heterogeneity, causing more than 17000 deaths in the US in the past year alone (1). A variety of treatment options, including surgery, neoadjuvant or adjuvant chemotherapy, immunotherapy, targeted therapy, and radiotherapy, provide competent tools for urologists to cure patients with BLCA (2). Nonetheless, most BLCA patients are not sensitive to these current treatments, which leads to high overall mortality (3–6). Hence, it is imperative to explore new prognostic biomarkers and treatment response predictors, which promoting individual precision treatment for BLCA.

Currently, the revolution of immune checkpoint blockades (ICB), including anti-PD-L1 or anti-PD-1 therapies, brings hope to patients with advanced BLCA (7). The response rate of ICB mainly depends on the tumour microenvironment (TME) (8). The TME is a comprehensive system consisting of tumour cells, tumour-infiltrating immune cells (TIICs), and an extracellular matrix. The categories and infiltration levels of TIICs, including CD8 T cells and Tregs, influence the activities of the anticancer immune response (9–11). The status and components of the TME are regulated by several mechanisms. One of the most important of these mechanisms is TGF-β (12–16).

The TGF-β pathway significantly regulates cancer biology, especially in anticancer immune responses (17). On the one hand, TGF-β suppresses tumour progression and strengthens TME homeostasis by inducing cancer cell apoptosis (18, 19). On the other hand, autocrine and paracrine TGF-β stimulates EMT, commonly mediated by the transcription factors SNAIL and SLUG, leading to resistance to anticancer treatments (17–19). TGF-β promotes immune evasion of cancer cells by suppressing the proliferation, differentiation, and immunocompetence of many immune cells, such as cytotoxic cells, DCs, and NK cells (16, 20–22). Consistently, activation of the TGF-β pathway indicated poor survival and resulted in resistance to ICB. Despite the prominent role of TGF-β in regulating multiple cancer processes, therapies targeting the TGF-β pathway have not been well explored (10). With the improvement of genomic sequencing methods, more and more genomic signatures have been created to predict the prognosis and therapeutic opportunities for cancers (8, 23, 24). In BLCA, Stojnev et al. explored the roles of three key components (TGF-β1, Smad2, and Smad4) of the canonical TGFβ pathway in predicting the prognosis (25). However, few studies have been conducted to systematically correlate the TGF-β signature with the tumour microenvironment phenotypes for BLCA.

This study, first established a novel TGF-β signature by integrating several independent BLCA cohorts, including the TCGA-BLCA and Xiangya cohorts. The TGF-β signature was closely related to TME characteristics and could predict several therapeutic opportunities for BLCA.

## Materials and Methods

### Data Sets Collection and Data Pre-Processing

#### Training Cohort

We downloaded TCGA-BLCA mRNA sequencing data (FPKM) and clinical data from The Cancer Genome Atlas (TCGA). The FPKM values were then switched into TPM values. The TCGA-BLCA cohort included 403 BLCA samples and 19 paired normal tissues.

#### Validation Cohorts

Xiangya cohort (internal validation set) was collected in our hospital. The Xinagya cohort included 57 BLCA samples sequenced by the BGISEQ-500 platform (BGI-Shenzhen, China) (GSE188715) (8, 23). The TPM values of the mRNA expression matrix were used to do the analysis. Another two external GEO cohorts, GSE32894 (224 samples) and GSE48075 (73 samples), with detailed survival information, were gathered from the Gene Expression Omnibus (GEO). The original RNA expression matrices of these two GEO cohorts were directly downloaded and eligible to be directly analysed. Three immunotherapy associated cohorts were collected, including IMvigor210 cohort, GSE78220, and GSE100797.

#### TGF-β Pathway Genes

Currently, TGF-β signature genes have not been well summarized. Therefore, we systematically searched all TGF-β-related genes from several common public databases with the following search terms: GO:0007179 from AmiGO 2 (http://amigo.geneontology.org/amigo/landing), TGF-beta from Ensembl Genome Brower (http://grch37.ensembl.org/index.html), BIOCARTA_TGFB_PATHWAY and KEGG_TGF_BETA_SIGNALING_PATHWAY from GSEA (http://www.gsea-msigdb.org/gsea/index.jsp). Eventually, a total of 225 TGF-β genes were collected (**Supplementary Table 1**).

Clinicopathologic information of these data sets is provided in **Supplementary Table 1**.

### Screening Differentially Expressed TGF-β Genes

We identified TGF-β DEGs between bladder cancer and normal tissues by using the empirical Bayesian approach of the limma R package. |log_2_ (fold change) | > 1 and the adjusted P-value < 0.05 were defined as standardization to determine significant TGF-β DEGs (26). Then, we used the ClusterProfiler R package to perform Gene Ontology (GO) analyses and Kyoto Encyclopedia of Genes and Genomes (KEGG) analyses based on the TGF-β DEGs (26). Furthermore, the protein–protein interaction network of those TGF-β DEGs was plotted by using the STRING database.

### Development and Validation of the TGF-β Risk Score

First, the prognostic TGF-β DEGs were screened by using univariate Cox analysis and survival R package in the TCGA-BLCA cohort. Second, we performed the least absolute shrinkage and selector operation (LASSO) regression on the prognostic TGF-β DEGs to reduce the dimensionality of high-dimensional data in the TCGA-BLCA cohort. The variables with relatively small contributions to the outcomes were given zero coefficients in the LASSO regression analysis. Then, the genes with nonzero coefficients were ultimately selected for multivariable Cox regression analysis and further construct the TGF-β risk score. The computation formula of the risk score is as follow:

Risk score = Σ *βi* * *RNAi*, where βi is the coefficient of the i-th gene in multivariable Cox regression analysis.

Then, all patients were classified into two groups (high-risk and low-risk score groups) based on the median of the TGF-β risk scores (23, 27, 28). To illustrate the survival difference between the high-risk and low-risk groups, Kaplan–Meier survival analysis was used to generate the survival curves. The prognostic significance of the TGF-β risk score was estimated by using the log-rank test. In addition, we plotted receiver operating characteristic (ROC) curves by using the ‘survival ROC’R package. We calculated the area under the curve (AUC) to assess the accuracy of the TGF-β risk score in predicting prognosis (26). We subsequently correlated the TGF-β risk score with the tumour stage and grade. Finally, the role of the TGF-β risk score was further validated in several independent BLCA validation sets, including the GSE32894, GSE48075, and Xiangya cohorts.

### Development and Validation of a Comprehensive Nomogram

We performed univariate and multivariate Cox regression analyses to screen the independent prognostic characters based on clinicopathologic characters and TGF-β risk score. Only 19 patients diagnosed with high tumour grade existed in the TCGA-BLCA cohort. As a result, the tumour grade was not included in the Cox survival analysis. Furthermore, we integrated the prognostic factors in univariate Cox analysis to construct a comprehensive nomogram to predict the prognosis of BLCA patients. The statistical performance of the comprehensive nomogram was evaluated by plotting ROC and calibration curves in the TCGA-BLCA cohort and several external validation cohorts.

### Defining the Immunological Characters in the BLCA TME

The anticancer immune response in the BLCA TME comprises several steps: tumour cells releasing cancer cell antigens (Step 1); tumour cells presenting cancer antigens to immune cells in the TME (such as dendritic cells) (Step 2); antigen-presenting cells (APCs) carrying antigens priming and activating the immune system in the TME (Step 3); the initiation of the immune system results in the release of related chemokines and cytokines, hence recruiting immune cells infiltrating the TME (Steps 4-5); and cytotoxic immune cells, such as natural killer T cells and CD8+ T cells, existing in the TME recognizing cancer cells (Step 6) and killing them (Step 7) (29). The vitality of seven steps decides on the fate of the tumour cells (8, 23). In addition, we calculated the levels of tumour-infiltrating immune cells (TIICs) by adopting several independent methods, including TIMER, Quan TIseq, TIP, XCell, Cibersort-ABS, and MCP-Counter, based on the RNA-seq data (30–36). Pre-existing anticancer immunity in the TME can be reflected by the T cell inflamed score (TIS), which can predict the clinical response to immune checkpoint block (ICB) (37). Then, we used ssGSEA to calculate the enrichment scores of several immunotherapy response-related pathways (8, 38). Finally, we screened and collected 20 inhibitory immune checkpoints, including PD-1, PD-L1, and CTLA-4. These immunological features in the TME have been well described in our previous studies (8, 23).

### Predicting the Molecular Subtypes in BLCA

BLCA is a highly heterogeneous tumour. The confirmation of the individual molecular subtype contributes to personalized precision medicine for BLCA patients. Thus, we have lucubrated molecular subtype systems in our previous study, including CIT, Lund, MDA, TCGA, Baylor, UNC, and Consensus subtypes (8, 39–43). We adopted ConsensusMIBC and BLCAsubtyping R packages to define individual subtypes. In addition, we collected 12 heterogeneous signatures of BLCA. We further correlated the TGF-β risk score with these molecular subtypes and BLCA-specific signatures. After all samples were preliminarily assigned into basal or luminal subtypes, we plotted ROC curves to evaluate the predictive accuracy of the TGF-β signature in evaluating the molecular subtypes.

### Prediction of the Clinical Sensitivities to Several Treatment Options for BLCA

Since chemotherapy is critical for terminal BLCA patients, we used the pRRophetic package to evaluate the clinical response to six common chemotherapy drugs (docetaxel, bleomycin, paclitaxel, camptothecin, vinblastine, and cisplatin) based on data of the Genomics of Drug Sensitivity in Cancer (GDSC) (https://www.cancerrxgene.org/) (44). We compared the difference in the 50% inhibitory concentration (IC50) of the six chemotherapeutics mentioned above between the high-risk and low-risk score groups. Targeted therapies, radiotherapy, and optional therapeutic schedules are also crucial. Therefore, several potential predictors related to the response of radiotherapy and targeted therapy were gathered from our previous studies (8, 23).

### Statistical Analysis

Data visualization and statistical analyses were conducted by using R software (Version: 4.0.5). We adopted Pearson or Spearman correlation analyses to ascertain the relationships between continuous variables. As for continuous variables fitting normal distribution between binary groups, t test was used. Otherwise, we used the Mann–Whitney U test. Univariate and multivariate Cox regression analysis were used to evaluating the correlation between the risk score and prognosis. We calculated the accuracy of the TGF-β risk score in predicting prognosis and molecular subtypes by plotting the ROC curves. Analyses with two-sided *P* less than 0.05 were considered statistically significant.

## Results

### Screening of TGF-β DEGs and Corresponding Functional Enrichment Analysis

Sixty TGF-β DEGs were screened between BLCA and normal tissues (**Supplementary Table 2**). As shown in **Supplementary Figures 1A, B**, 24 TGF-β genes were over-expressed, while the 36 remaining TGF-β genes were downregulated in BLCA. For the GO_BP analysis, these TGF-β DEGs were enriched in several pathways, including the threonine kinase signalling pathway, TGF-β receptor signalling pathway, and cellular response to TGF-β stimulus (**Supplementary Figure 1C**). For the GO_CC analysis, these TGF-β DEGs were enriched in transcription regulator complex, RNA polymerase II transcription regulator complex, and phosphatase complex. For the GO_MF analysis, DEGs were enriched in transforming growth factor-beta binding, SMAD binding, and cytokine binding. Moreover, KEGG analyses demonstrated that TGF-β DEGs were also enriched in the TGF-β signalling pathway, Hippo signalling pathway, and bladder cancer (**Supplementary Figure 1D**). As expected, the TGF-β signalling pathway was distinctly the most common enrichment pathway of these DEGs. The PPI network analysis results indicated that these TGF-β DEGs were closely related to each other (**Supplementary Figure 1E**).

### Development and Validation of TGF-β Risk Score for BLCA

**Figure 1:**
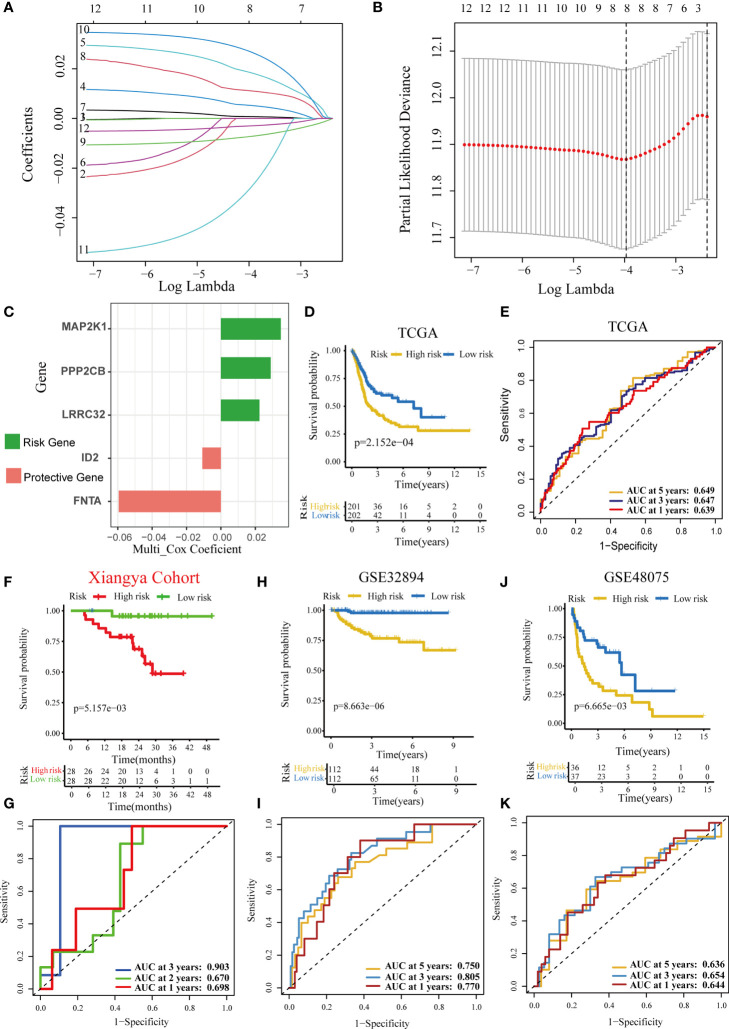
Development and validation of a TGF-β risk score in several BLCA cohorts. **(A)** LASSO coefficients of 12 prognostic TGF-β genes in the TCGA-BLCA cohort. **(B)** Cross-validation for turning parameter selection *via* minimum criteria in the LASSO regression model. **(C)** The coefficients of five optimal TGF-β genes included into the final multivariate Cox analysis. Green indicates the risk gene, while red represents the protective gene. **(D)** Kaplan–Meier analysis of OS for TGF-β risk score in the TCG-BLCA cohort. **(E)** ROC curves of the TGF-β risk score in predicting OS in the TCG-BLCA cohort. **(F, G)** Validation of the TGF-β risk score in the Xiangya cohort. **(H, I)** Validation of the TGF-β risk score in GSE32894. **(J, K)** Validation of TGF-β risk score in the GSE48075.

First, we identified 12 prognostic genes from the 60 TGF-β DEGs in TCGA-BLCA cohort (**Supplementary Table 2**). Second, we performed a LASSO regression based on these 12 prognostic genes and screened eight genes with nonzero coefficients (**Figures 1A, B**). The minimum lamda was 0.019. Eventually, we identified five optimal TGF-β genes (MAP2K1, PPP2CB, LRRC32, ID2, and FNTA) based on the Akaike information criterion (AIC). The results of univariate Cox regression analyses based on these five optimized genes included in the risk score model is shown in **Supplementary Table 3A**. We then calculated the TGF-β risk score by performing multivariate Cox regression on these five genes. **Figure 1C** and **Supplementary Table 3B** displayed the coefficients of these five genes. In TCGA-BLCA cohort, patients were assigned into high-risk and low-risk score groups based on the median risk score. Patients with high-risk score had poorer OS than patients with low-risk score (**Figure 1D**). Meanwhile, the accuracy of the TGF-β risk score in predicting OS was acceptable (**Figure 1E**). To adjust the bias caused by clinicopathological heterogeneity, we performed further subgroup analyses based on different clinicopathological features. As shown in **Supplementary Figure 2**, patients with high-risk score had poorer prognosis regardless of the subgroups. In addition, we successfully validated the role of the TGF-β risk score in three independent validation cohorts, including the Xiangya cohort, GSE32894, and GSE48075 (**Figures 1F**–**K**). In these validation cohorts, patients with high-risk score had poorer OS. Especially in the Xiangya cohort, the accuracy of the TGF-β risk score in predicting the three-year OS was more than 0.9 (**Figure 1G**).

### Development and Validation of a Comprehensive Nomogram Based on Clinicopathological Characteristics and TGF-β Risk Score

**Figure 2:**
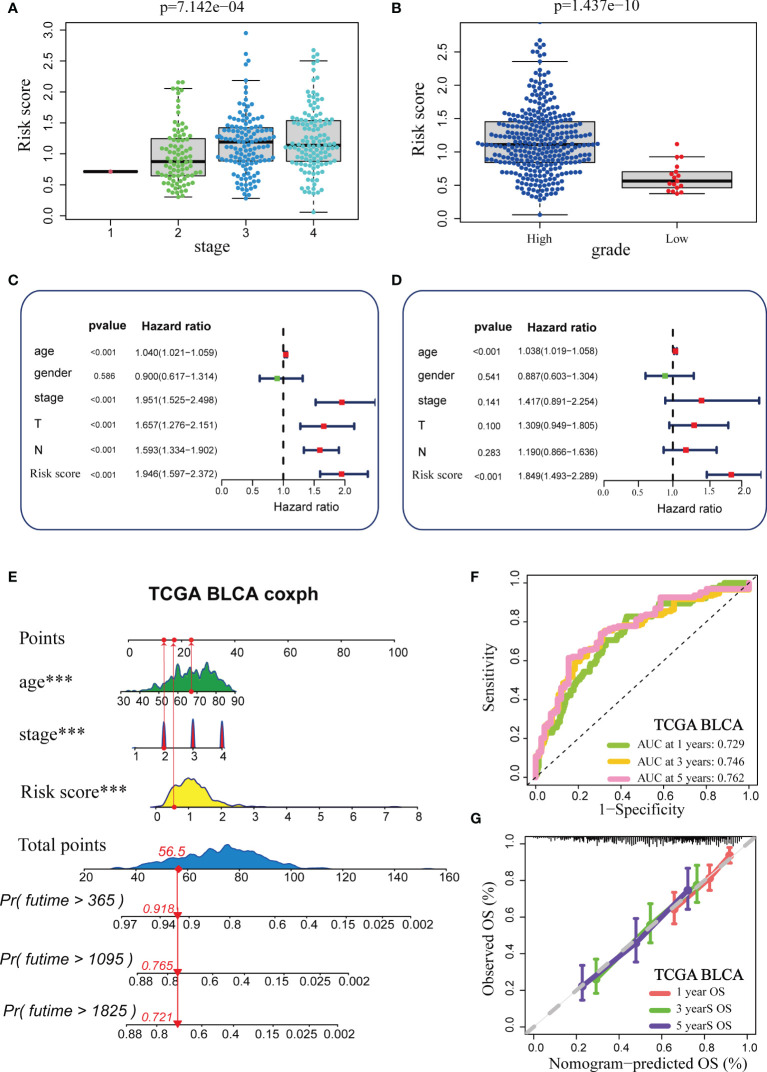
Construction and validation of a comprehensive nomogram in the TCGA-BLCA cohort. **(A, B)** Relationships between TGF-β risk score, tumour stage, and tumour grade in the TCGA-BLCA cohort. **(C, D)** Results of univariate and multivariate Cox analysis. **(E)** Development of the nomogram based on stage, age and TGF-β risk score. **(F)** The ROC curves of the nomogram. **(G)** The Calibration plots for the nomogram. (***P < 0.001).

We analysed the correlations between TGF-β risk score and tumour grade, tumour stage. As expected, patients with advanced grades and stages had higher risk scores, which was in accord with the adverse prognostic role of the TGF-β risk score (**Figures 2A, B**). We successfully validated these results in several external cohorts (**Supplementary Figure 3**). In addition, we analysed the relationships between the risk score and histological subtypes, muscle invasiveness, and metastasis (**Supplementary Figure 4**). We found a higher risk score in cancers with nonpapillary histology and muscle invasiveness (**Supplementary Figures 4A, B, D, F**). Patients with metastasis had higher risk score compared with patients without metastasis (**Supplementary Figures 4C, E**). The significant difference was not significant, which may be attributed to the small number of patients with metastasis. The univariate Cox analysis results based on several common clinicopathological characteristics showed that tumour stage, age, and TGF-β risk score were prognostic factors (**Figure 2C**). We subsequently performed multivariate Cox analysis and further confirmed the independent prognostic role of the TGF-β risk score (**Figure 2D**). As a result, the TGF-β risk score was a robust prognostic predictor for BLCA. Then, we integrated the TGF-β risk score with two other prognostic factors (stage and age) to exploit a comprehensive nomogram (**Figure 2E**). The accuracy of the nomogram in predicting 1-, 3-, and 5-year overall survival was 0.729, 0.746, and 0.762, separately, which was obviously higher than the accuracy of the TGF-β risk score (**Figure 2F**). The nomogram-predicted OS was consistent with the real OS as shown in the calibration curves (**Figure 2G**).

**Figure 3:**
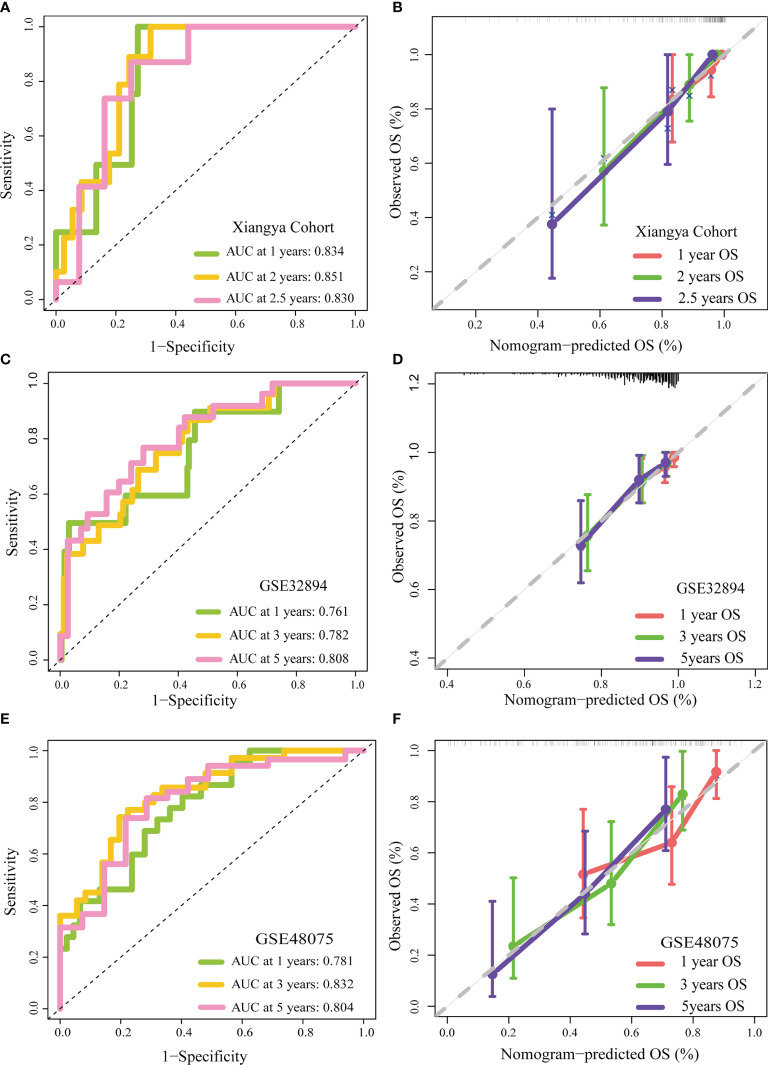
External validation of the TGF-β risk score. **(A, B)** Role of the TGF-β risk score in the Xiangya cohort. **(C, D)** Role of the TGF-β risk score in GSE32894. **(E, F)** Role of the TGF-β risk score in GSE48075.

We appraised and validated the ability of the nomogram in predicting prognosis among three external BLCA cohorts, including the Xiangya cohort, GSE32894, and GSE48075. In the Xiangya cohort, the accuracy in predicting 1-, 2-, and 2.5-year OS was 0.834, 0.851, and 0.830, separately (**Figure 3A**). The nomogram-predicted OS was highly similar to the real OS (**Figure 3B**). Similarly, we observed the same results in GSE32894 and GSE48075 (**Figures 3C**–**F**).

### TGF-β Risk Score Predicted the Immune Phenotypes of the TME and the Therapeutic Response to ICB

**Figure 4:**
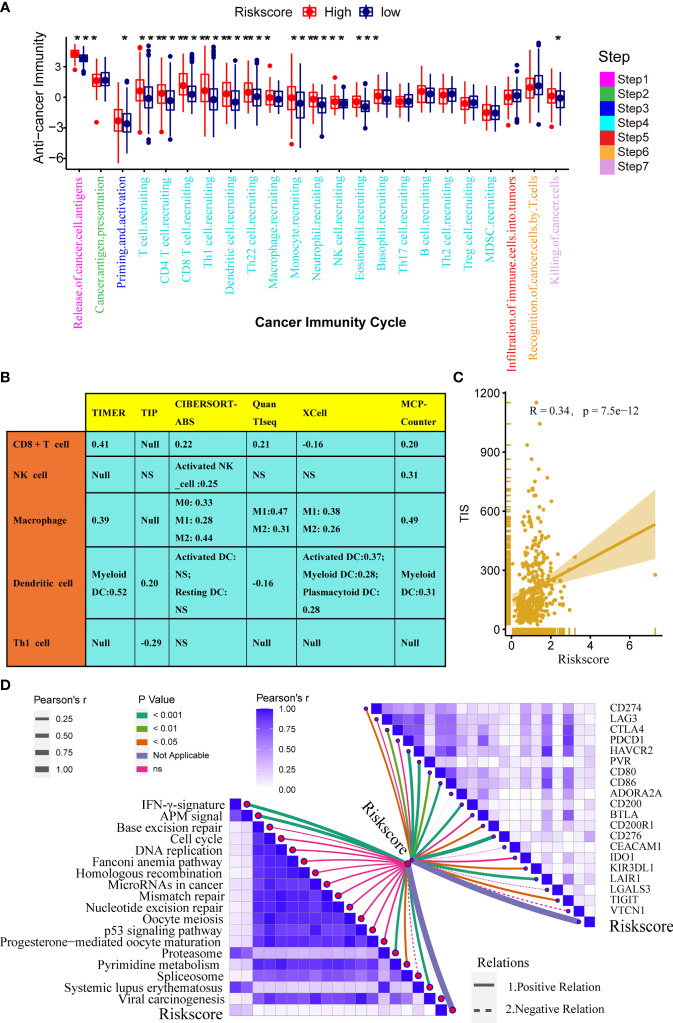
Relationships between the TGF-β risk score and the tumour immune microenvironment characteristics. **(A)** Differences between the high- and low-risk score groups with respect to the activities of the cancer immunity cycles. **(B)** Relationships between the TGF-β risk score and the levels of several critical TIICs (CD8+ T cells, NK cells, macrophages, Th1 cells, and DCs) in six independent algorithms (right panel). **(C)** Relationships between the TGF-β risk score and the T cell inflamed score (TIS). **(D)** Relationships between the TGF-β risk score and the enrichment of ICB response related pathways (left panel), and relationships between the TGF-β risk score and immune checkpoints (right panel). (*P < 0.05; **P < 0.01; ***P < 0.001; NS, not significant).

The immune status of the TME influences the fate of cancer cells and predicts sensitivity to ICB. First, we analysed the relationships between the risk score and the activities of the anticancer immunity cycles. Activities of several steps, such as the release activities of cancer cell antigens, priming, and activation, were significantly upregulated in patients with high-risk score (**Figure 4A**). This indicated that the anticancer immune response of the TME in patients with higher risk scores was more activated and immunogenic. Subsequently, the activities of several steps that recruited TIICs, such as CD8 + T cells, Th1 cells, NK cells, and DCs, were also significantly upregulated in patients with higher risk score. Therefore, the anticancer killing activity was consistently higher in patients with high risk score. Besides, we verified that the infiltration levels of TIICs, including CD8 + T cells, macrophages, NK cells, DCs, and Th1 cells, were positively correlated with the risk score in six independent algorithms (**Figure 4B**). Relationships between the TGF-β risk score and the levels of other immune cells or stromal cells in six separate algorithms are shown in **Supplementary Table 4**. These data revealed that a high-risk score predicted a hot TME status in BLCA. In line with the common sense that a hot TME was more sensitive to ICB, the TGF-β risk score positively correlated with the TIS (Spearman R = 0.34) (**Figure 4C**). Furthermore, the risk score was positively related to the expression of 20 inhibitory immune checkpoints and the scores of several immunotherapy response related pathways, including the IFN-γ signature, DNA repair, and base excision repair (**Figure 4D**). Correlations between the risk score and immune checkpoints are shown in **Supplementary Table 5**. Notably, the risk score positively correlated with CD274 (PD-L1) (P < 0.05). Overall, a high TGF-β risk score indicated an inflamed TME phenotype and may be more sensitive to ICB. To adjust the bias caused by clinicopathological heterogeneity, we performed further subgroup analyses based on different clinicopathological features. As expected, the correlations between TGF-β risk score and TME immune status were highly consistent in all the subgroups (**Supplementary Figures 5**–**10**). Therefore, the TGF-β risk score was a robust predictive biomarker for TME immune phenotypes.

In addition, we analysed the correlations between the risk score and TME stromal components, such as tumour fibroblasts and several stromal signatures. Tumour fibroblasts, as essential stromal components of the TME, were reported to have a close connection with the TGF-β pathway. Consistently, our results displayed a remarkable positive correlation between the TGF-β risk score and cancer-associated fibroblasts in several independent algorithms (**Supplementary Table 4**). In addition, the risk score positively correlated with many stromal signatures, such as pan-F-TBRS (panfibroblast TGFβ response signature) and EMT (epithelial-mesenchymal transition) signatures (**Supplementary Figure 11**).

### TGF-β Risk Score Predicted Molecular Subtypes and Guided Precision Medicine for BLCA

**Figure 5:**
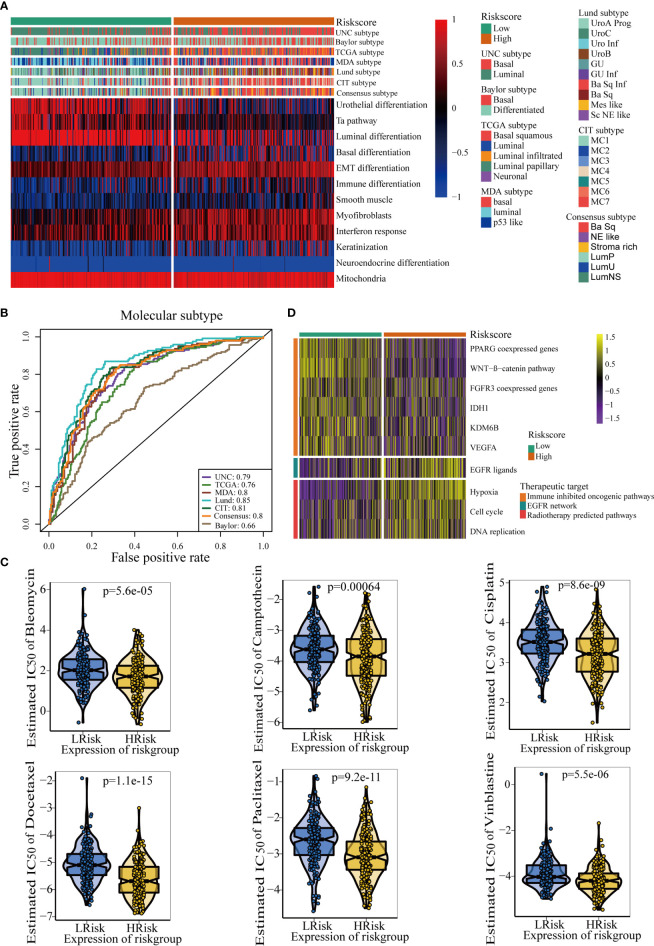
The TGF-β risk score predicted molecular subtypes and promoted individual precision medicine for BLCA. **(A)** Correlations between TGF-β risk score, bladder cancer signatures, and molecular subtypes from seven algorithms. **(B)** Predictive accuracy of the TGF-β risk score for molecular subtypes in several different methods. **(C)** There are differences in the therapeutic responses to six chemotherapy drugs. **(D)** Relationships between TGF-β risk score and the enrichment scores of several therapeutic signatures.

The molecular subtypes could guide individual precision treatment for BLCA. Therefore, we first correlated the TGF-β risk score with several traditional molecular subtype classification systems, including the UNC, TCGA, MDA, Lund, CIT, Consensus, and Baylor subtypes. As shown in **Figure 5A**, cancers with higher risk score were more likely to be basal subtype, featured by higher enrichment scores of basal differentiation and immune activation. However, cancers with lower risk score were more likely to be luminal subtype, featured by higher enrichment scores of luminal differentiation and urothelial differentiation. Furthermore, the results of ROC curves demonstrated that the accuracy of the TGF-β risk score in predicting the molecular subtypes of UNC, TCGA, MDA, Lund, CIT, Consensus, and Baylor systems were 0.79, 0.76, 0.80, 0.85, 0.81, 0.80, and 0.66, respectively (**Figure 5B**).

The IC50 of six common chemotherapy drugs was lower in patients with high risk score (**Figure 5C**). Similarly, the enrichment scores of EGFR ligands and radiotherapy response-related pathways were higher in patients with high risk score (**Figure 5D**). These data revealed that patients in the high risk score groups may benefit more from chemotherapy, EGFR-targeted therapy, and radiotherapy. However, several oncogenic signatures which supressed the anticancer immunity were enriched in patients with low-risk score (**Figure 5D**). Therefore, blocking these oncogenic pathways may improve the prognosis of patients with low TGF-β risk scores.

### Roles of TGF-β Risk Score in the Xiangya Cohort and External Validation Cohorts

**Figure 6:**
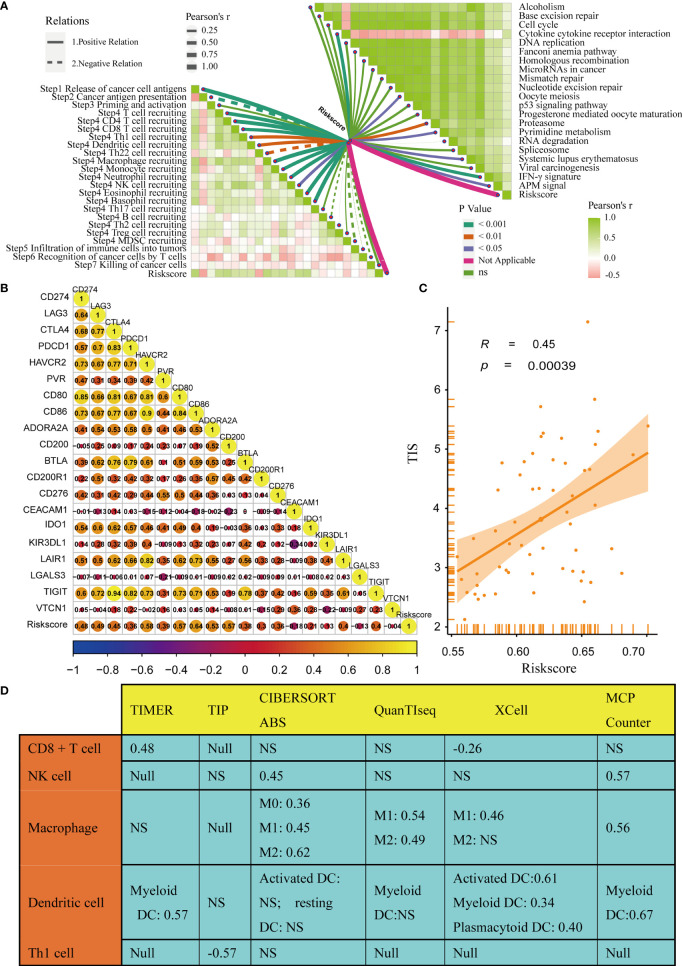
Relationships between the TGF-β risk score and TME immune characters in the Xiangya Cohort. **(A)** Relationships between TGF-β risk score and activities of the cancer immunity cycles (left panel); Relationships between TGF-β risk score and the enrichment scores of ICB response related pathways (right panel). **(B)** Relationships between the risk score and several immune checkpoints. **(C)** Correlation between the risk score and the T cell inflamed score (TIS). **(D)** Correlations between the risk score and the levels of several TIICs in six independent algorithms. NS, not significant.

**Figure 7:**
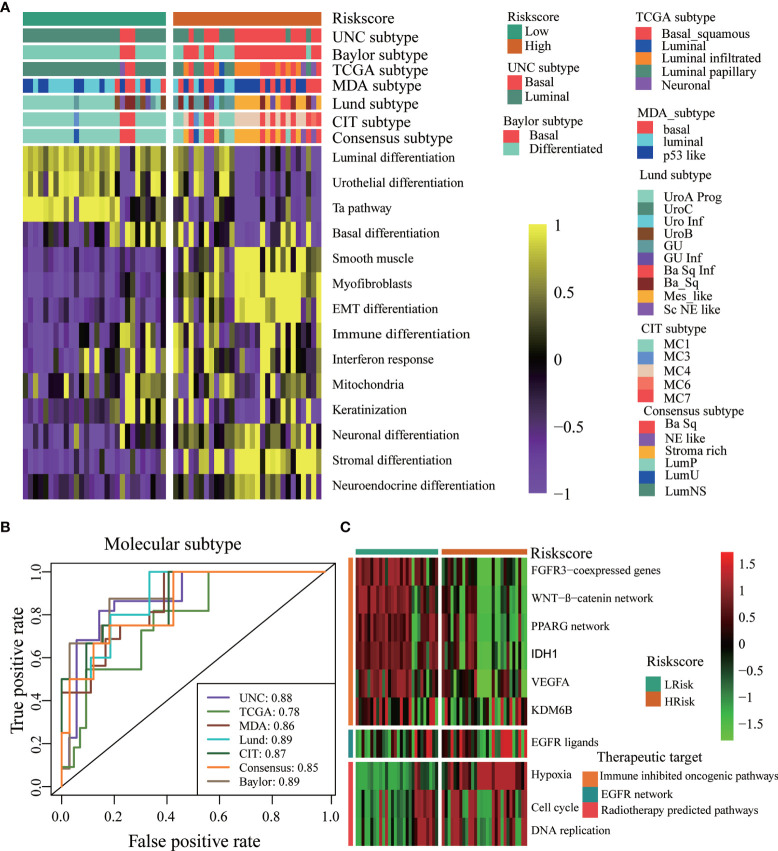
The TGF-β risk score predicted molecular subtypes and treatments opportunities in the Xiangya Cohort. **(A)** The risk score accurately stratified the molecular subtypes in different algorithms. **(B)** Accuracy of the risk score in predicting molecular subtypes in different algorithms. **(C)** Associations between the TGF-β risk score and the enrichment scores of several therapeutic signatures.

We further explored the associations between the TGF-β risk score and TME immune phenotypes, molecular subgroups, and therapeutic response in our Xiangya cohort. First, we proved that the TGF-β risk score was positively related to many anticancer immunity steps (**Figure 6A** left panel). In addition, the risk score positively correlated with the levels of several TIICs, including CD8 + T cells, macrophages, and DCs, in six independent algorithms (**Figure 6D**). The TGF-β risk score was also positively related to the enrichment of ICB response-related signatures, the expression of 20 immune checkpoints, and TIS (**Figures 6A** right panel, **B, C**). The results of the Xiangya cohort proved that a high risk score indicated an inflamed phenotype. Then, we further validated the role of TGF-β risk score in differentiating the molecular subtypes in the Xiangya cohort (**Figures 7A, B**). Finally, we confirmed the closed associations between the risk score and clinical responses to several treatment options, including targeted therapies, EGFR therapies, and radiotherapy (**Figure 7C**). It was apparent that patients with high-risk scores may benefit more from radiotherapy and EGFR targeted treatment. However, patients with low-risk scores may benefit more from targeted treatments, such as blocking out the WNT-β-catenin networks or FGFR3 pathways (**Figure 7C**). All of these results were well-validated in two other cohorts, GSE32894 and GSE48075 (**Supplementary Figures 12**, **13**). Finally, we correlated the risk score with therapeutic response and prognosis of patients in three immunotherapy associated cohorts (**Supplementary Figure 14**). In the IMvigor210 cohort, we did not observe relationship between the risk score and prognosis in the whole cohort or in three immune phenotypes. Most of patients in IMvigor210 cohort received chemotherapy before immunotherapy, which may diminish the correlations between the TGF-β risk score and therapeutic response and prognosis. In the GSE100797 cohort, patients in the high risk score group had better prognosis and higher ICB response rates, though the statistical difference was not significant. Similar results were observed in the GSE78220 cohort.

## Discussion

It is widely known that the TGF-β pathway is a critical factor for tumour development. However, TGF-β had two-sided effects (45). From one point of view, TGF-β protected tumour cells from malignant evolution. The inverse aspect stated that TGF-β modulated processes such as cell invasion and immune suppression, which cancer cells may take advantage of (17). In addition, the expression and activation of TGF-β could be considered potential targets for antitumor therapy (46). With revealing the mechanisms of TGF-β in regulating tumour biology, more and more attention has been paid to the therapeutic ability of TGF-β (17).

Currently, the TGF-β signalling pathway is extensively used for prognosis prediction in various tumours, such as ovarian cancer, hepatocellular carcinoma, non-small-cell lung cancer (NSCLC), and colorectal cancer (47–50). Previous works have shown that TGF-β is critical for development and progression of bladder cancer. For instance, TGF-β-induced transgelins could promote bladder cancer metastasis (51), and the TGF-β-related miR-1305/TGF-β2/smad3 pathway was shown to help circRIP2 accelerate bladder cancer development (52). These studies indicated a close correlation between TGF-β and bladder cancer. However, little work has systematically contributed to exploring the role of TGF-β-related signatures in regulating TME characteristics and predicting prognosis in BLCA. Thus, we thoroughly explored TGF-β in BLCA in this study to compensate for this gap. We screened various differentially expressed TGF-β-related genes. Then, we selected five vital genes among them to construct a TGF-β signature according to the standard of our method. These genes included FNTA, PPP2CB, LRRC32, ID2, and MAP2K1.

These five genes were correlated with tumour progression. FNTA was regulated by scaffold association factor B (SAFB) to promote RAS membrane association, which was buttressed to exploit a rational approach to anticancer therapy (53). It was remarkable that our team once intensively studied FNTA and found its association with BLCA. The androgen receptor promoted BLCA by regulating the expression level of circRNA-FNTA (54); PPP2CB was reported as protein phosphatase 2A. This enzyme regulated various of cellular biological processes, including tumour development (55). It is one of the indicators for 8p suppressor gene (56). LRRC32 was upregulated on CD4^+^ CD25^+^ FOXP3^+^ Treg cells; thus, it was always considered a Treg-specific activation marker (57). GARP is regarded as a receptor for LAP/latent TGF-β (58). The GARP and GARP-ligand signalling pathways was demonstrated to be a target for cancer immunotherapy (58). ID2 plays a critical role in the survival of aggressive cancer cells (59) and positively modulates the activity of HIF2α in glioblastoma (60). MAP2K1, also known as MEK1, plays a critical role in inhibiting the progression of histiocytic neoplasms (61). Based on these five genes, we integrated multiple BLCA datasets (TCGA-BLCA, GSE32894, GSE48075), in addition to the Xiangya cohort, to develop and validate a novel TGF-β risk score.

TGF-β signalling was proven to be a dominant suppressor of adaptive and innate immune responses during tumour progression (62). We regarded the risk score as reflecting the characteristics of the TME from several aspects. First, we verified that the TGF-β risk score predicted the survival and immune characteristics in the TME. As displayed in our plots, patients with higher risk scores always had lower OS rates, higher tumour grade and stage. Second, we screened the difference in the enrichment scores of immunotherapy response-related signatures between the high-risk and low-risk score groups. The IFN-γ signature, APM signal, and base excision repair were obviously enriched in patients with high risk score. Chemotherapy significantly improved the survival for patients with advanced BLCA (63). Therefore, the development of accurate indicators for chemotherapy is extremely urgent. Interestingly, we found that patients with high-risk scores might benefit more from chemotherapy.

Here, the TGF-β risk score had positive correlations with the enrichment scores of several anticancer immunity cycles, TIS, and TIICs. As a result, the activity of pre-existing anticancer immunity in the TME was higher in patients with high risk score (37). Based on the theory of immune checkpoints inhibiting anticancer immunity in the TME (64), we consistently correlated the risk score with the expression of representative immune checkpoints, including CD274, CTLA4, and CD276. The correlation was positive and significant between the risk score and ICI. The results showed that a tumour TME with a higher risk score indicated a better effect of immunotargeted therapy and more sensitivity to immunotherapy. However, inadequacy and doubt still existed. The results also showed that the risk score positively correlated with macrophages (M2), which were considered a signal to inhibit anticancer immunity (**Figure 4B**). Hence, the pre-existing anticancer activities of patients with high risk scores may be restrained by the higher level of tumour infiltrated macrophages (M2) and the over-expression of many inhibitory immune checkpoints (such as CD274 and LAG3). Treatments, especially ICB, were suitable for patients with high-risk scores whose anticancer immunity was suppressed to a certain extent (8). In contrast, patients with low-risk scores always had lower TIS and lower expression of immune checkpoints, which indicated that they might not be suitable for ICB.

Anil Korkut et al. presented a pan-cancer analysis of genomic alterations that regulated TGF-β signalling in a large sample set. The results identified mutation hotspots in the TGF-β superfamily, which indicated potential biomarkers for further therapeutic and cancer diagnostic studies. Their work demonstrated the significance and prospect of a thorough investigation of TGF-β (65). Several studies focusing on the prognostic prediction of TGF-β have been published. Slavica Stojnev et al. evaluated the associations of TGF-β1, Smad4, and Smad2 with clinicopathologic characteristics and prognosis in urothelial bladder cancer (25). We compared our work with these previous works. First, our key genes were screened from numerous TGF-β-related genes with more comprehensive algorithms, such as the lasso algorithm and Akaike information criterion. Second, we constructed a five-gene signature and correlated it with molecular subtypes, TME immune characteristics and therapeutic opportunities. Third, we validated our model in several external cohorts. Of note, we did not perform immunohistochemical staining, which could be considered a flaw. The construction and validation of scoring models has been a core of ceaseless studies in oncology research. Numerous risk models related to EMT, immune TME, and hypoxia have been lucubrated and reported with the potential to predict prognosis and guide precision medicine for BLCA (23, 25, 65–68). It was remarkable that our previous work clarified thought in screening key genes, constructing a gene model, associating the model with immune characteristics, and evaluating the accuracy for predicting prognosis and treatment response. In the current study, we adopted a similar thought and obtained results as expected.

However, drawbacks inevitably existed in several aspects of our study. First, all of our outcomes, including the construction of the signature and the nomogram, were based on public data and clinical cohorts. Although we also validated these results in the Xiangya cohort, molecular mechanisms and experiments *in vivo* or *in vitro* may be necessary. Second, only two external cohorts and the Xiangya cohort (only 57 samples included) contributed to the verification of our outcomes. More prospective data are urgently needed for further validation of the TGF-β signature due to our clinical data limitations. Third, the clinical appliance of the TGF-β signature needs more exploration. The risk model was developed based on the RNAseq data. As a result, qPCR may be a more suitable method to calculate the risk score during clinical practice. In addition, we will design a corresponding genetic testing kit based on these five genes of the risk model. Fourth, we dogmatically consider the median of the TGF-β risk score as the cut-off value in all analyses, which was needed to be further validated.

In summary, we took one small step forwards in the sphere of exploring rational prognostic predictors for BLCA. We developed and validated a well-grounded TGF-β risk score based on realistic clinical data. The TGF-β risk score was proven to stratify the clinical outcomes and TME phenotypes of BLCA. It also indicated the sensitivity of several treatment options for BLCA. We found that patients with high-risk scores may benefit more from ICB, radiotherapy, chemotherapies, and EGFR-targeted therapy. However, patients with low-risk scores may be more suitable to receive other targeted therapies, including blocking out the FGFR3 or WNT-β-catenin networks.

## Data Availability Statement

Publicly available datasets were analyzed in this study. These data can be found at: TCGA-BLCA, https://portal.gdc.cancer.gov/; GSE48075: https://www.ncbi.nlm.nih.gov/geo/query/acc.cgi?acc=GSE48075; GSE32894: https://www.ncbi.nlm.nih.gov/geo/query/acc.cgi?acc=GSE32894; GSE78220: https://www.ncbi.nlm.nih.gov/geo/query/acc.cgi?acc=GSE78220; GSE100797: https://www.ncbi.nlm.nih.gov/geo/query/acc.cgi?acc=GSE100797; IMvigor210: http://research-pub.gene.com/IMvigor210CoreBiologies/; The Xiangya cohort has been uploaded to GEO database (GSE188715: https://www.ncbi.nlm.nih.gov/geo/query/acc.cgi?acc=GSE188715). The critical R codes were provided within the **Supplementary Material**.

## Ethics Statement

The collection of the Xiangya cohort was approved by the Ethics Committee of the Xiangya Hospital of Central South University.

## Authors Contributions

Conception and design: ZL, JH, ZO, BO, and XZ. Provision of study materials or patients: TQ, XL, and YY. Collection and assembly of data: JH and XZ. Data analysis and interpretation: JH, ZO, XZ, ZY, and JC. Manuscript writing: ZL, ZO, JH, XZ, and TQ. Final approval of manuscript: All authors.

## Funding

This work was supported by the grants from the Science and Technology Joint Fund Project in Guizhou Province [LH(2016)7386], Hunan Provincial Natural Scientific Foundation [2020JJ5916], Guizhou Provincial Education Department Youth Science and Technology talent Growth Project [KY(2018)172], and the National Natural Science Foundation of China [82070785, 81873626, 81902592].

## Conflict of Interest

The authors declare that the research was conducted in the absence of any commercial or financial relationships that could be construed as a potential conflict of interest.

## Publisher’s Note

All claims expressed in this article are solely those of the authors and do not necessarily represent those of their affiliated organizations, or those of the publisher, the editors and the reviewers. Any product that may be evaluated in this article, or claim that may be made by its manufacturer, is not guaranteed or endorsed by the publisher.

## Supplementary Material

Supplementary Figure 1 Identification of differentially expressed TGF-β genes (TGF-β DEG genes) and functional analyses of these genes. **(A)** Sixty differentially expressed TGF-β genes were identified between BLCA and normal tissues. **(B)** The TGF-β DEG genes were shown in volcano plot. **(C, D)** GO, and KEGG analyses of the TGF-β DEG genes. **(E)** The PPI network of the TGF-β DEG genes.

Supplementary Figure 2 Survival analysis based on different clinicopathological characteristics.

Supplementary Figure 3 Correlations between the TGF-β risk score and clinicopathologic features in external validation sets.

Supplementary Figure 4 Associations between the TGF-β risk score and histological subtypes, muscle invasiveness, and metastasis.

Supplementary Figure 5 Correlations between the TGF-β risk score and tumour immune microenvironment characteristics in the ‘High stage’ subgroup.

Supplementary Figure 6 Correlations between the TGF-β risk score and tumour immune microenvironment characteristics in the ‘Low stage’ subgroup.

Supplementary Figure 7 Correlations between the TGF-β risk score and the tumour immune microenvironment characteristics in the ‘female’ subgroup.

Supplementary Figure 8 Correlations between the TGF-β risk score in tumours and the immune microenvironment characteristics in the ‘male’ subgroup.

Supplementary Figure 9 Correlations between the TGF-β risk score and the tumour immune microenvironment characteristics in the ‘Younger’ subgroup.

Supplementary Figure 10 Correlations between the TGF-β risk score in tumours and the immune microenvironment characteristics in the ‘Older’ subgroup.

Supplementary Figure 11 Correlations between TGF-β risk score and TME stromal components, including EMT1, EMT2, EMT3, and Pan-F-TBRS.

Supplementary Figure 12 Validation of the TGF-β risk score in the GSE32894 cohort. **(A)** TGF-β risk score correlated with the activities of the cancer immunity cycles. **(B)** The risk score correlated with the enrichment scores of ICB response related pathways. **(C)** The risk score correlated with the immune checkpoints. **(D)** The risk score accurately stratified the molecular subtypes in several different algorithms. **(E)** Accuracy of the risk score in predicting the molecular subtypes in several different algorithms. **(F)** The risk score correlated with the enrichment scores of several therapeutic signatures.

Supplementary Figure 13 Validation of the TGF-β risk score in the GSE48075 cohort. **(A)** TGF-β risk score correlated with the activities of the cancer immunity cycles. **(B)** The risk score correlated with the enrichment scores of ICB response related pathways. **(C)** The risk score correlated with the immune checkpoints. **(D)** The risk score accurately stratified the molecular subtypes in several different algorithms. **(E)** Accuracy of the risk score in predicting the molecular subtypes in several different algorithms. **(F)** The risk score correlated with the enrichment scores of several therapeutic signatures.

Supplementary Figure 14 Associations between the TGF-β risk score and therapeutic response of immunotherapy in three cohorts.

Supplementary Table 1 TGF-β genes and detailed information of four BLCA cohorts applied in our analysis.

Supplementary Table 2 Sixty differentially expressed TGF-β genes between BLCA and normal tissues.

Supplementary Table 3
**(A)** Coefficients of 5 optimal candidate genes included in the TGF-β risk score with univariate Cox regression analysis. **(B)** Coefficients of 5 optimal candidate genes included in the TGF-β risk score algorithm.

Supplementary Table 4 Correlations between the risk score and the infiltration levels of TIICs in six independent algorithms.

Supplementary Table 5 Correlations between the risk score and immune checkpoints.
