# Supplementary figures and images for: A Novel TGF-β Risk Score Predicts the Clinical Outcomes and Tumour Microenvironment Phenotypes in Bladder Cancer
Source: Front Immunol. 2021 Dec 17;12:791924. doi: 10.3389/fimmu.2021.791924 (PMC8718409; doi:10.3389/fimmu.2021.791924)

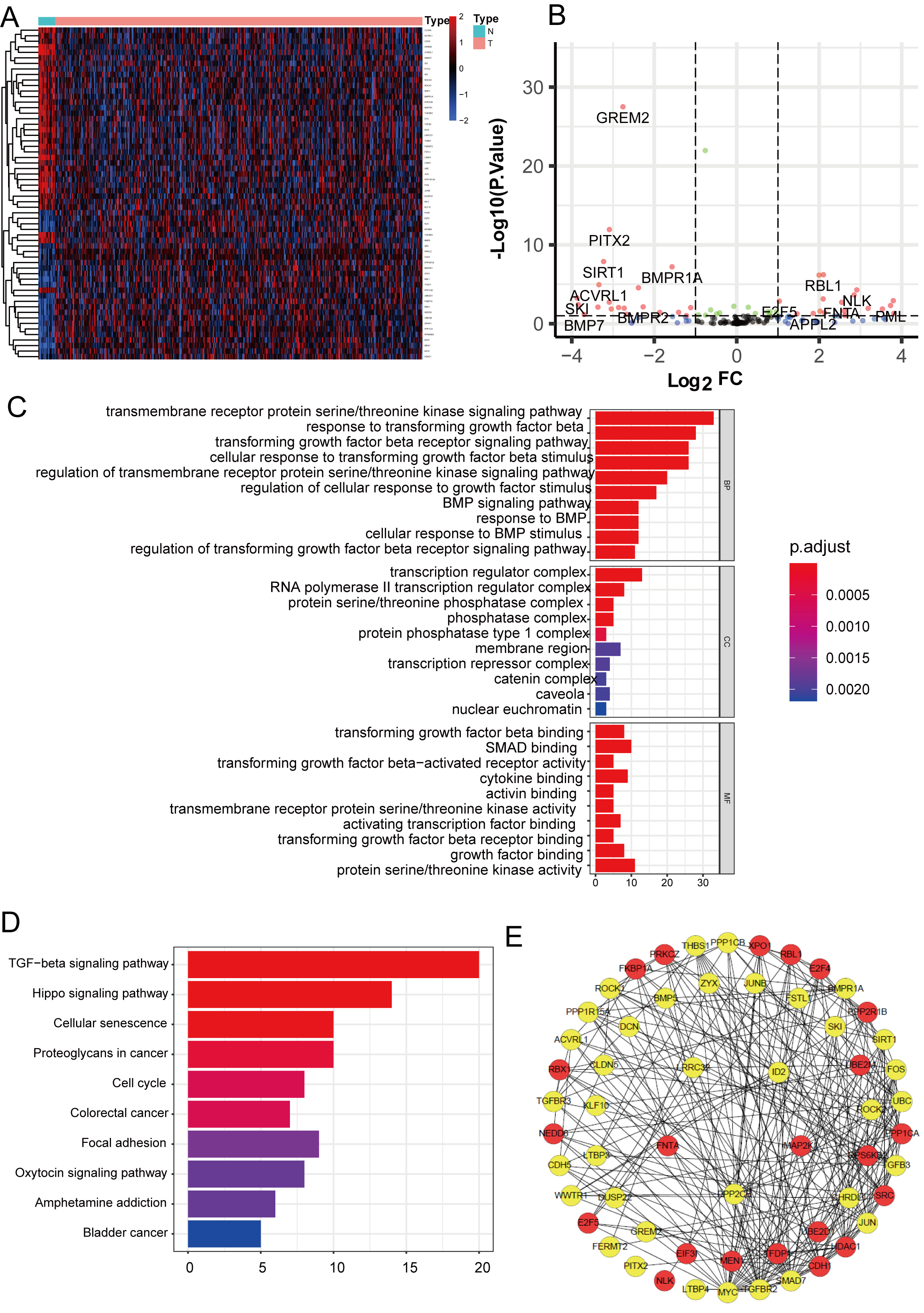

Supplement: Supplementary Figure 1 — Identification of differentially expressed TGF-β genes (TGF-β DEG genes) and functional analyses of these genes. (A) Sixty differentially expressed TGF-β genes were identified between BLCA and normal tissues. (B) The TGF-β DEG genes were shown in volcano plot. (C, D) GO, and KEGG analyses of the TGF-β DEG genes. (E) The PPI network of the TGF-β DEG genes. [file Image_1.tif]

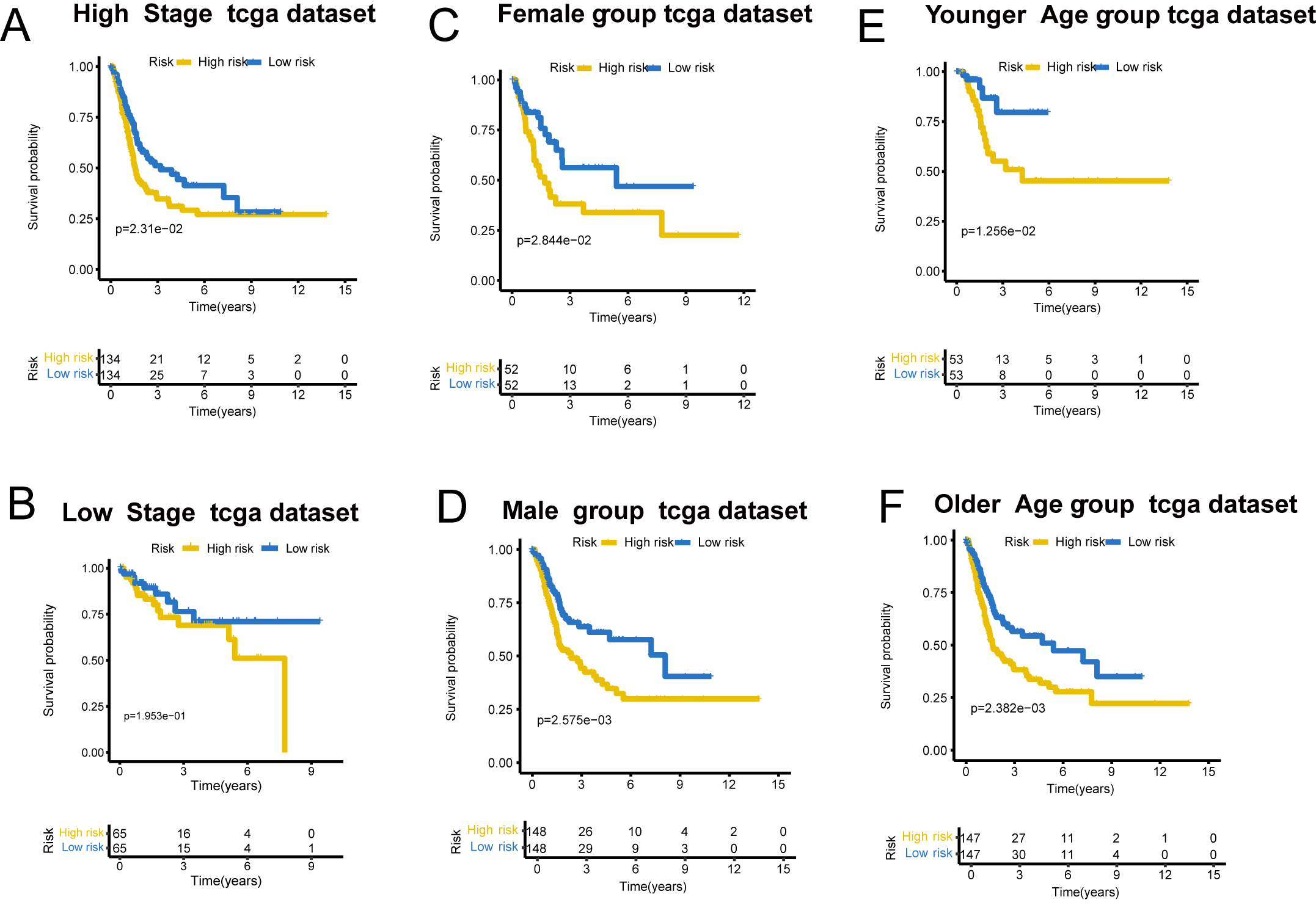

Supplement: Supplementary Figure 2 — Survival analysis based on different clinicopathological characteristics. [file Image_2.tif]

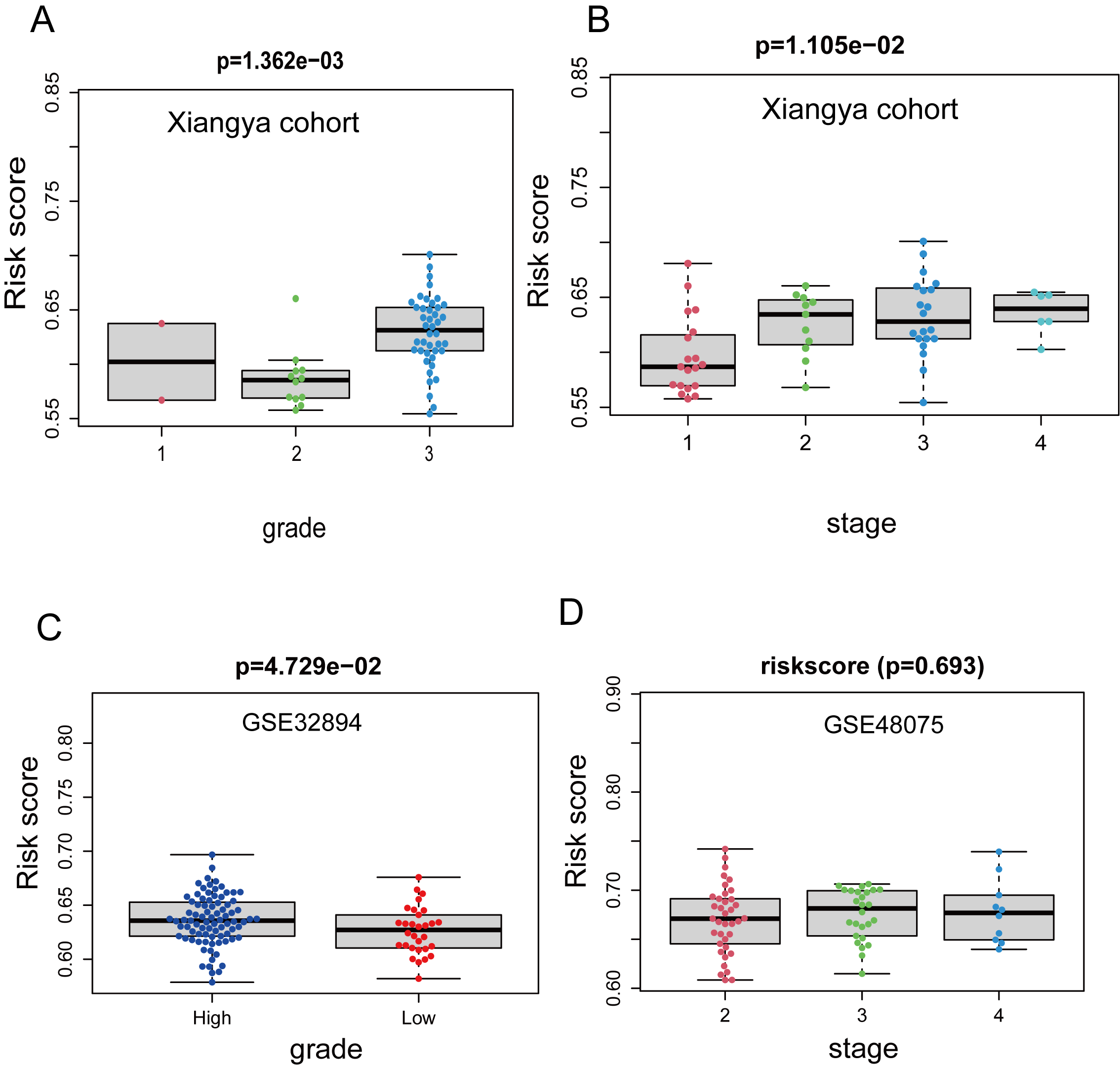

Supplement: Supplementary Figure 3 — Correlations between the TGF-β risk score and clinicopathologic features in external validation sets. [file Image_3.tif]

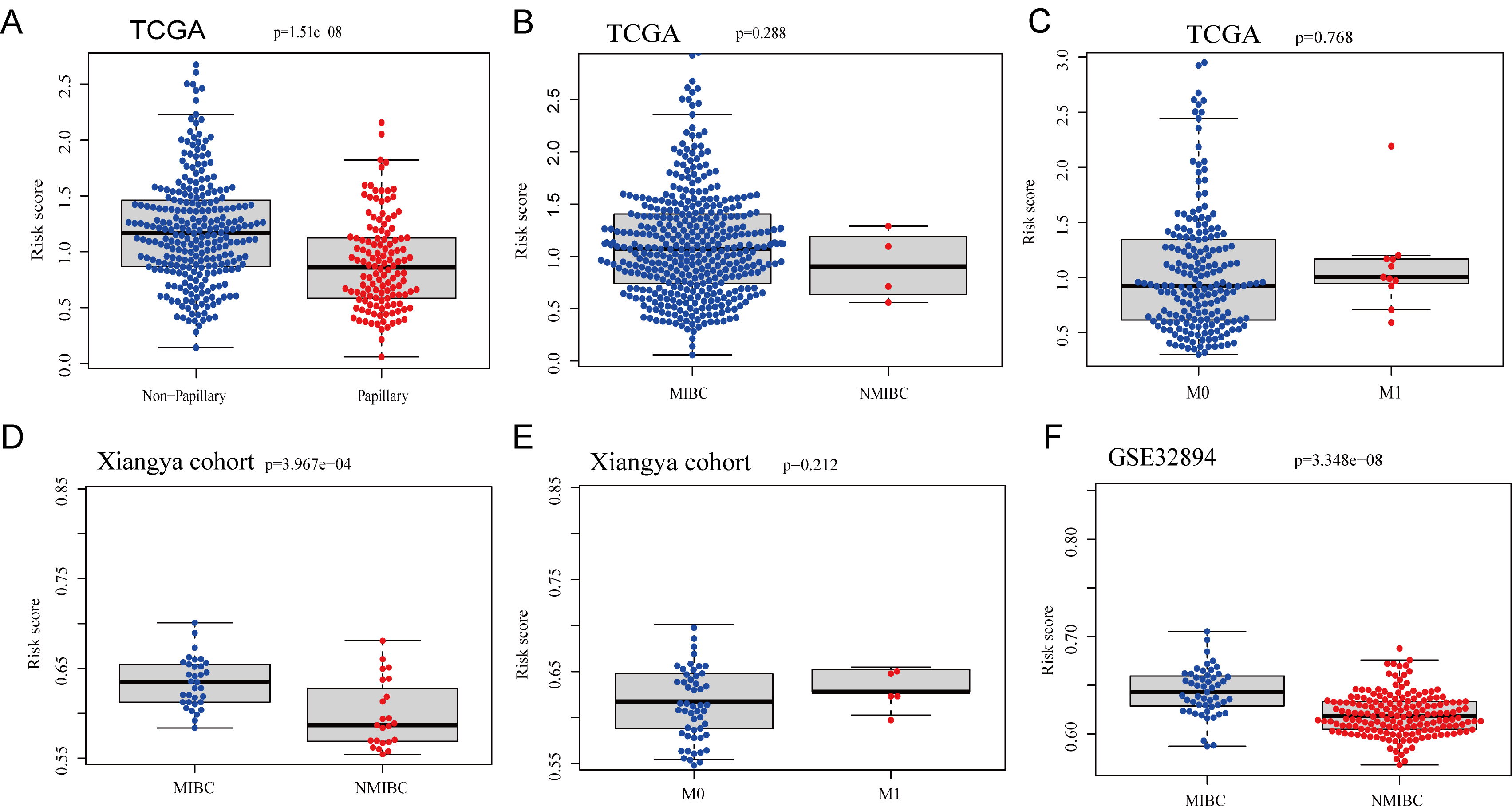

Supplement: Supplementary Figure 4 — Associations between the TGF-β risk score and histological subtypes, muscle invasiveness, and metastasis. [file Image_4.tif]

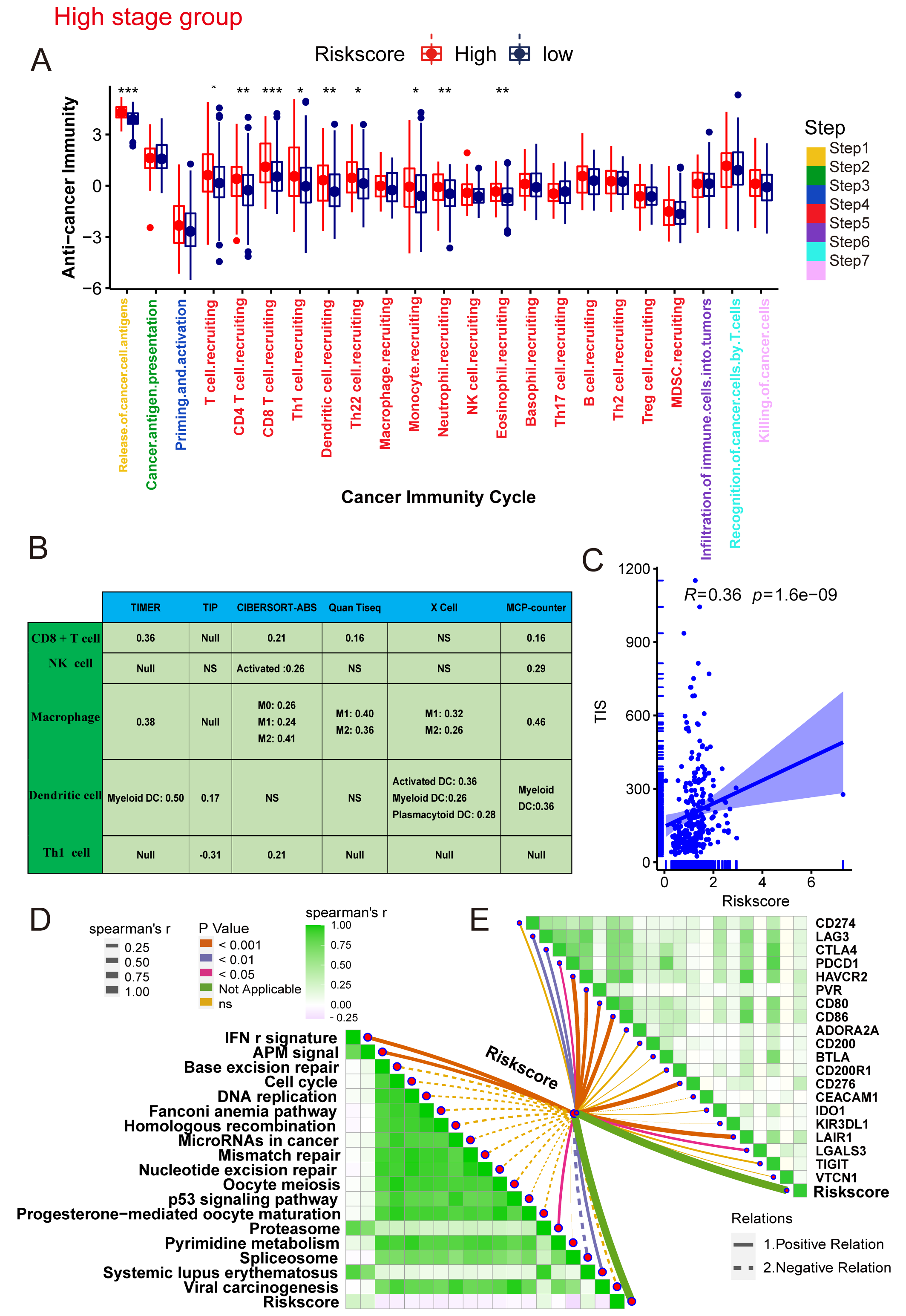

Supplement: Supplementary Figure 5 — Correlations between the TGF-β risk score and tumour immune microenvironment characteristics in the ‘High stage’ subgroup. [file Image_5.tif]

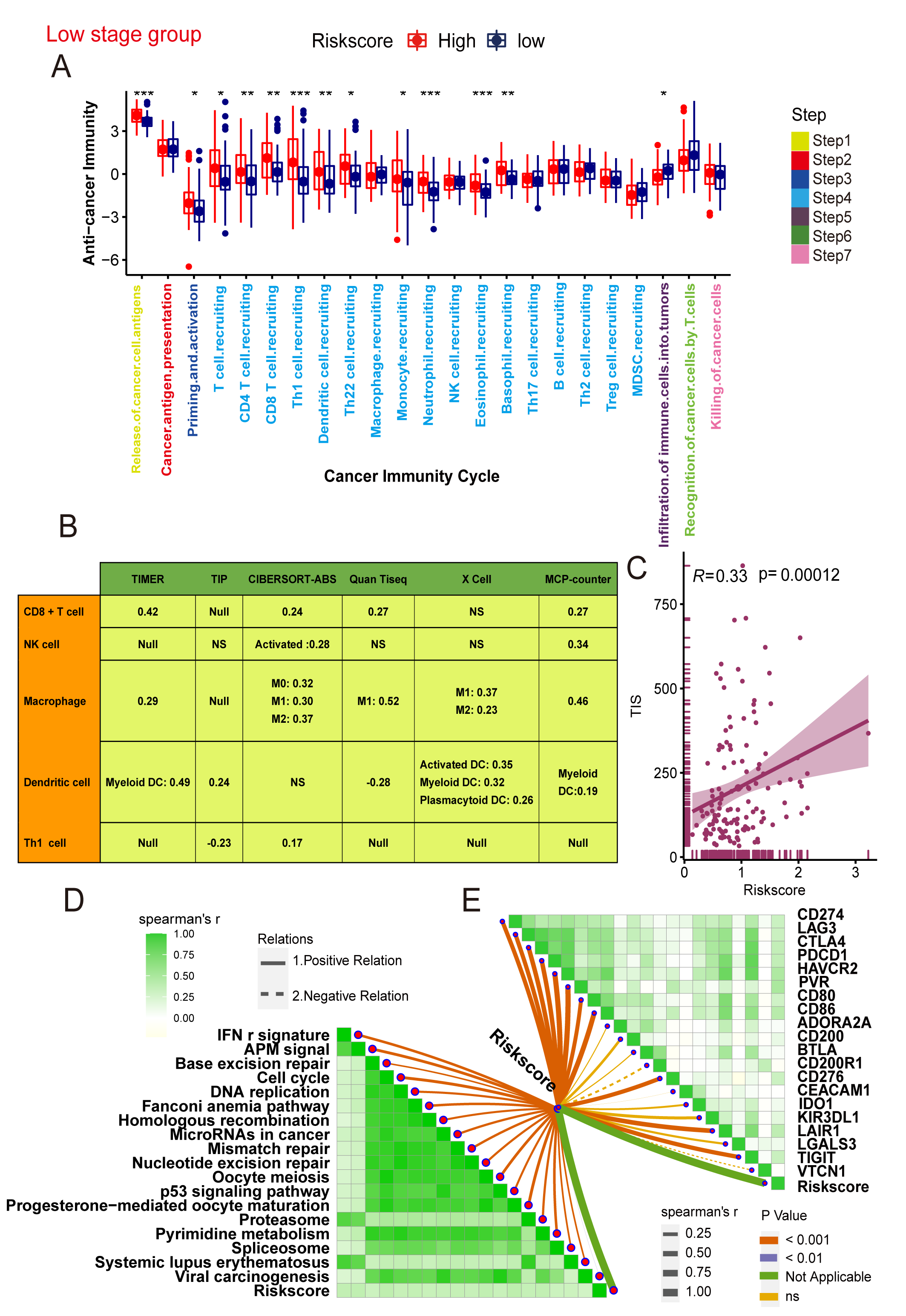

Supplement: Supplementary Figure 6 — Correlations between the TGF-β risk score and tumour immune microenvironment characteristics in the ‘Low stage’ subgroup. [file Image_6.tif]

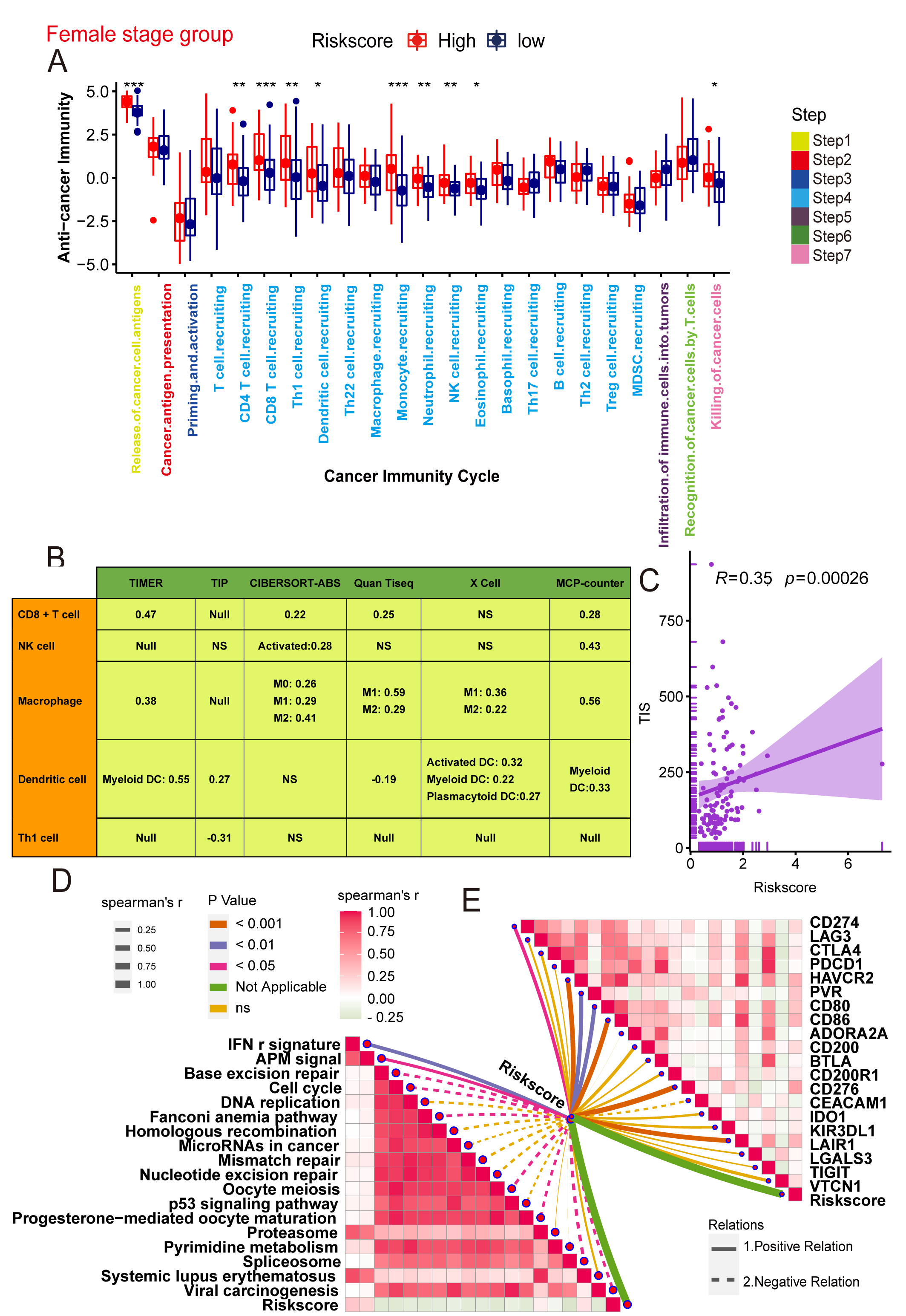

Supplement: Supplementary Figure 7 — Correlations between the TGF-β risk score and the tumour immune microenvironment characteristics in the ‘female’ subgroup. [file Image_7.tif]

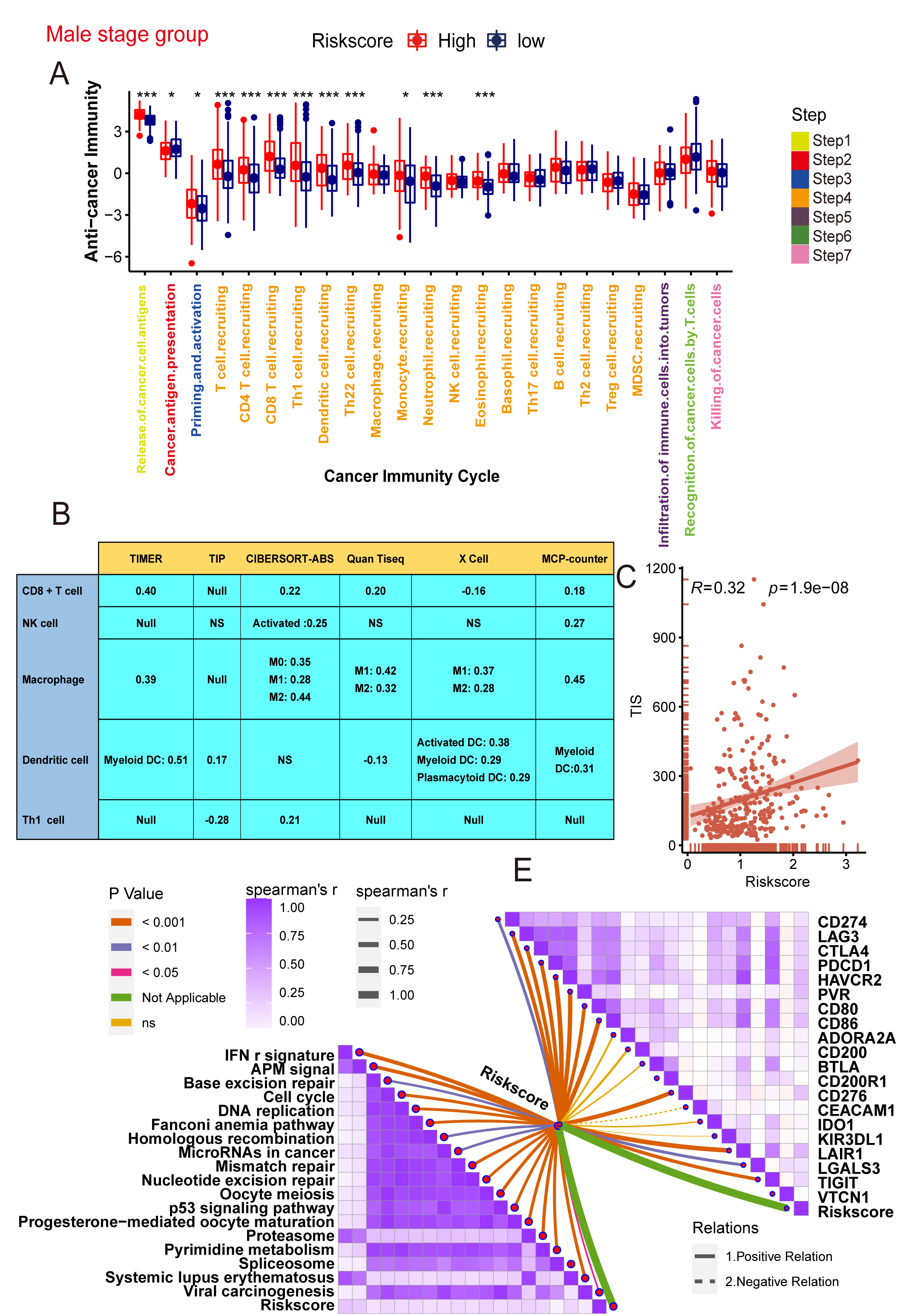

Supplement: Supplementary Figure 8 — Correlations between the TGF-β risk score in tumours and the immune microenvironment characteristics in the ‘male’ subgroup. [file Image_8.tif]

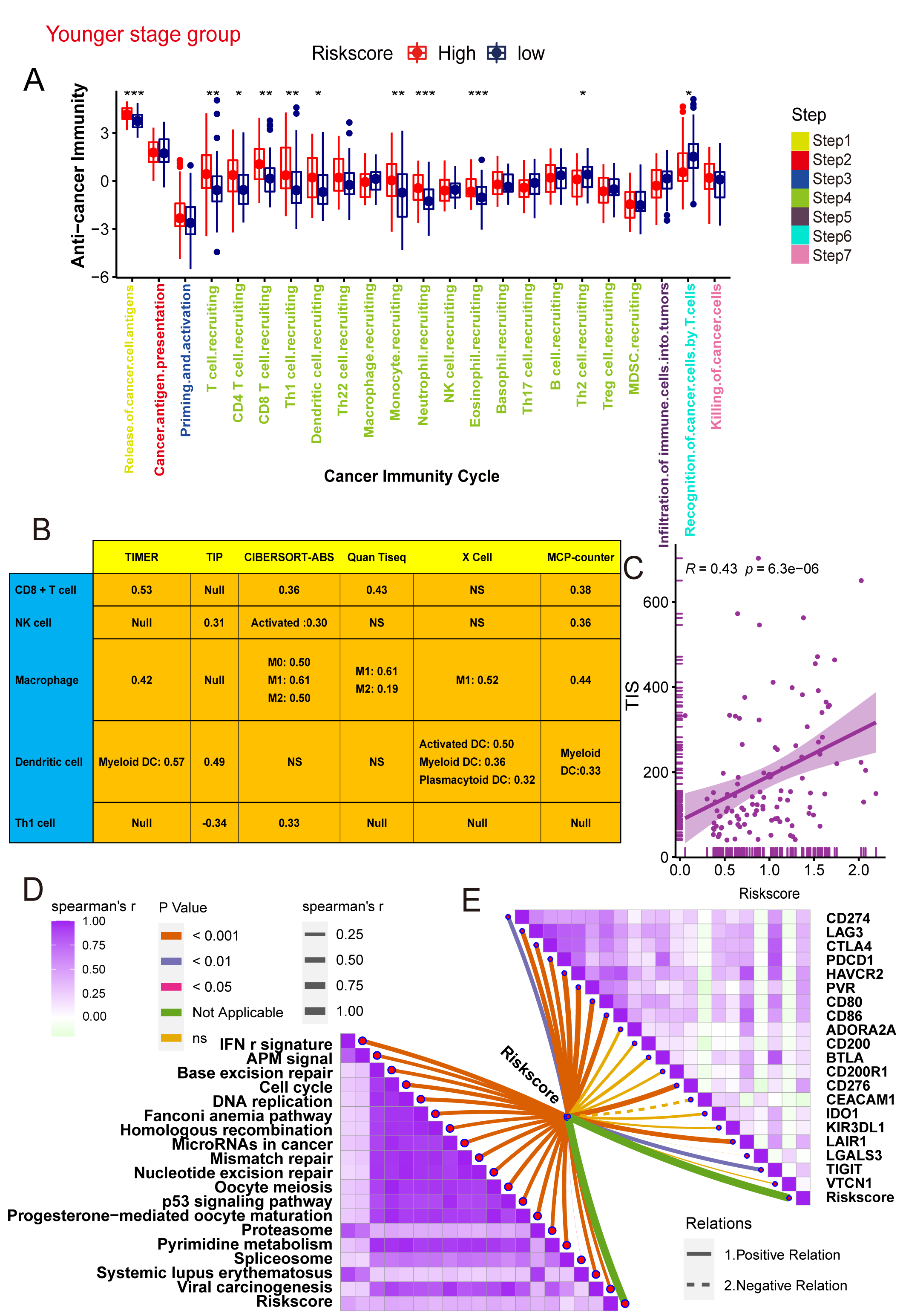

Supplement: Supplementary Figure 9 — Correlations between the TGF-β risk score and the tumour immune microenvironment characteristics in the ‘Younger’ subgroup. [file Image_9.tif]

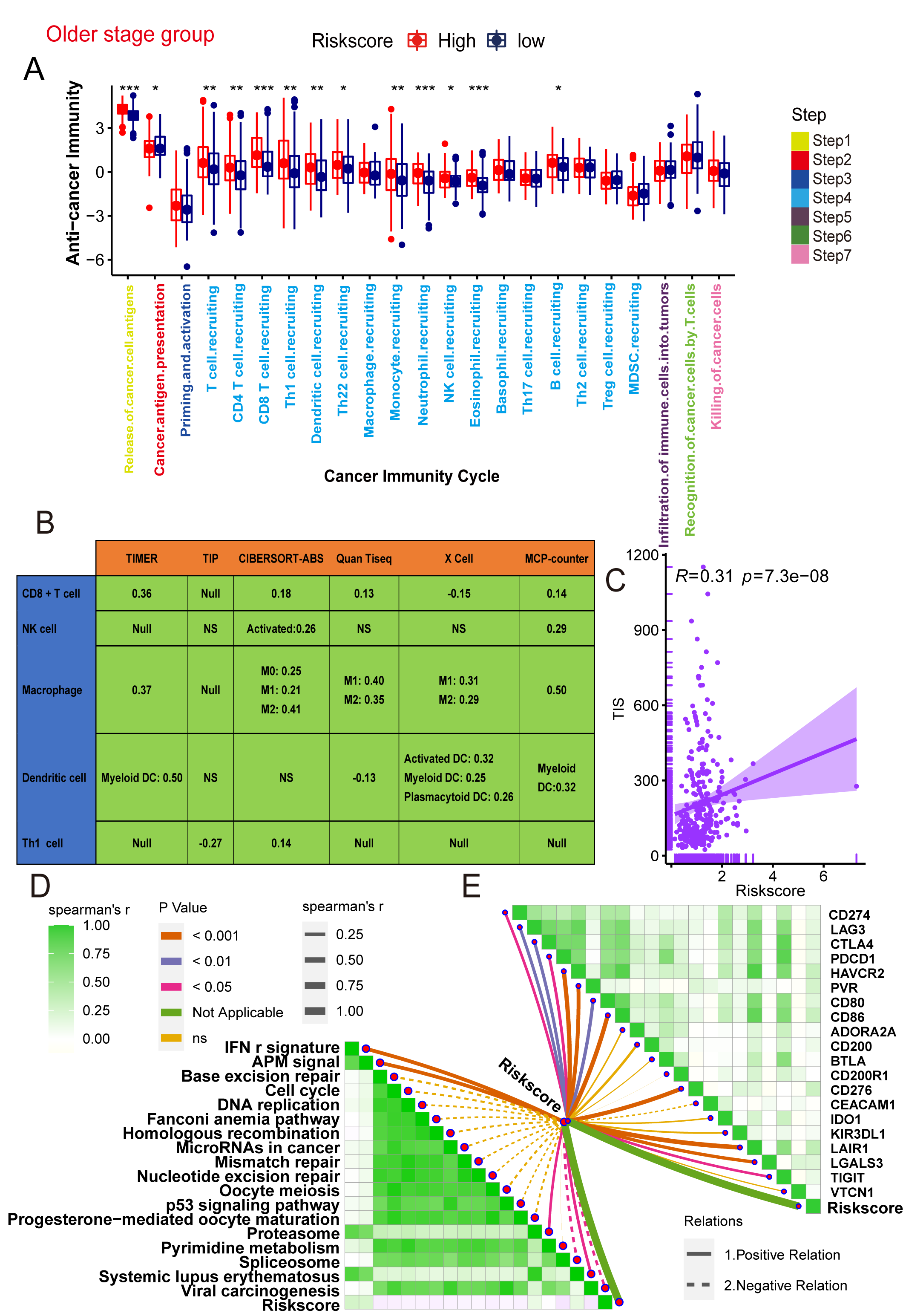

Supplement: Supplementary Figure 10 — Correlations between the TGF-β risk score in tumours and the immune microenvironment characteristics in the ‘Older’ subgroup. [file Image_10.tif]

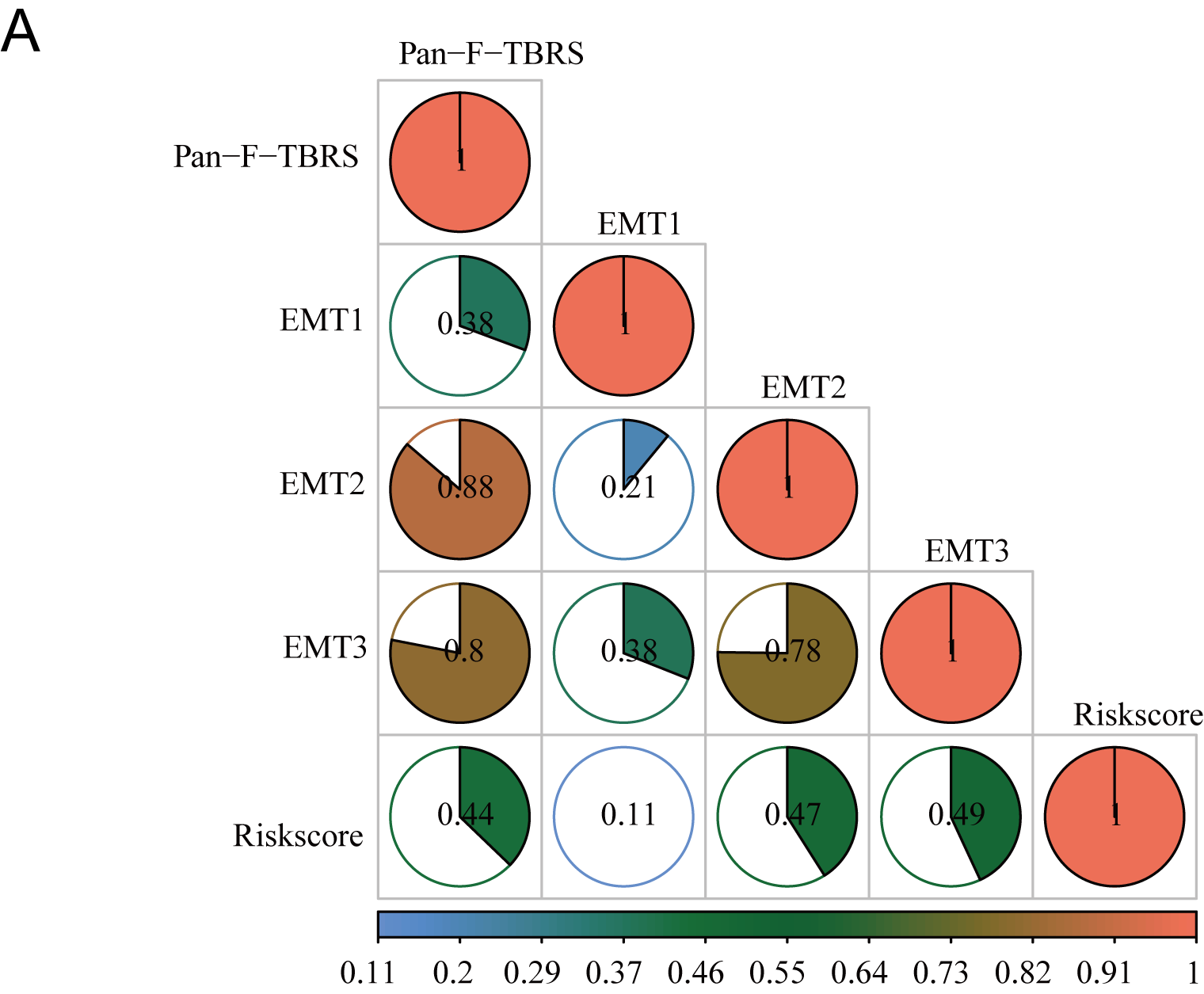

Supplement: Supplementary Figure 11 — Correlations between TGF-β risk score and TME stromal components, including EMT1, EMT2, EMT3, and Pan-F-TBRS. [file Image_11.tif]

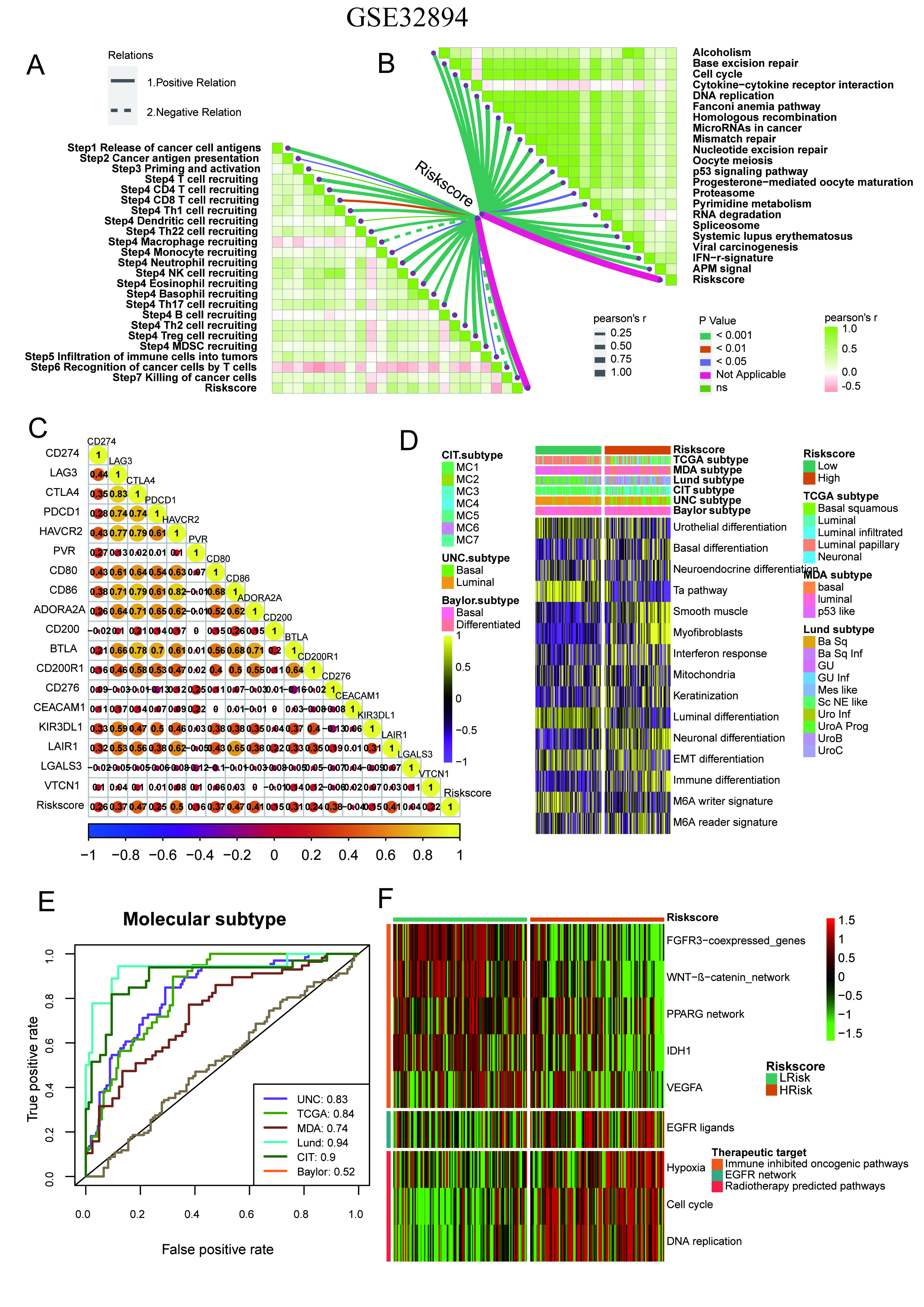

Supplement: Supplementary Figure 12 — Validation of the TGF-β risk score in the GSE32894 cohort. (A) TGF-β risk score correlated with the activities of the cancer immunity cycles. (B) The risk score correlated with the enrichment scores of ICB response related pathways. (C) The risk score correlated with the immune checkpoints. (D) The risk score accurately stratified the molecular subtypes in several different algorithms. (E) Accuracy of the risk score in predicting the molecular subtypes in several different algorithms. (F) The risk score correlated with the enrichment scores of several therapeutic signatures. [file Image_12.tif]

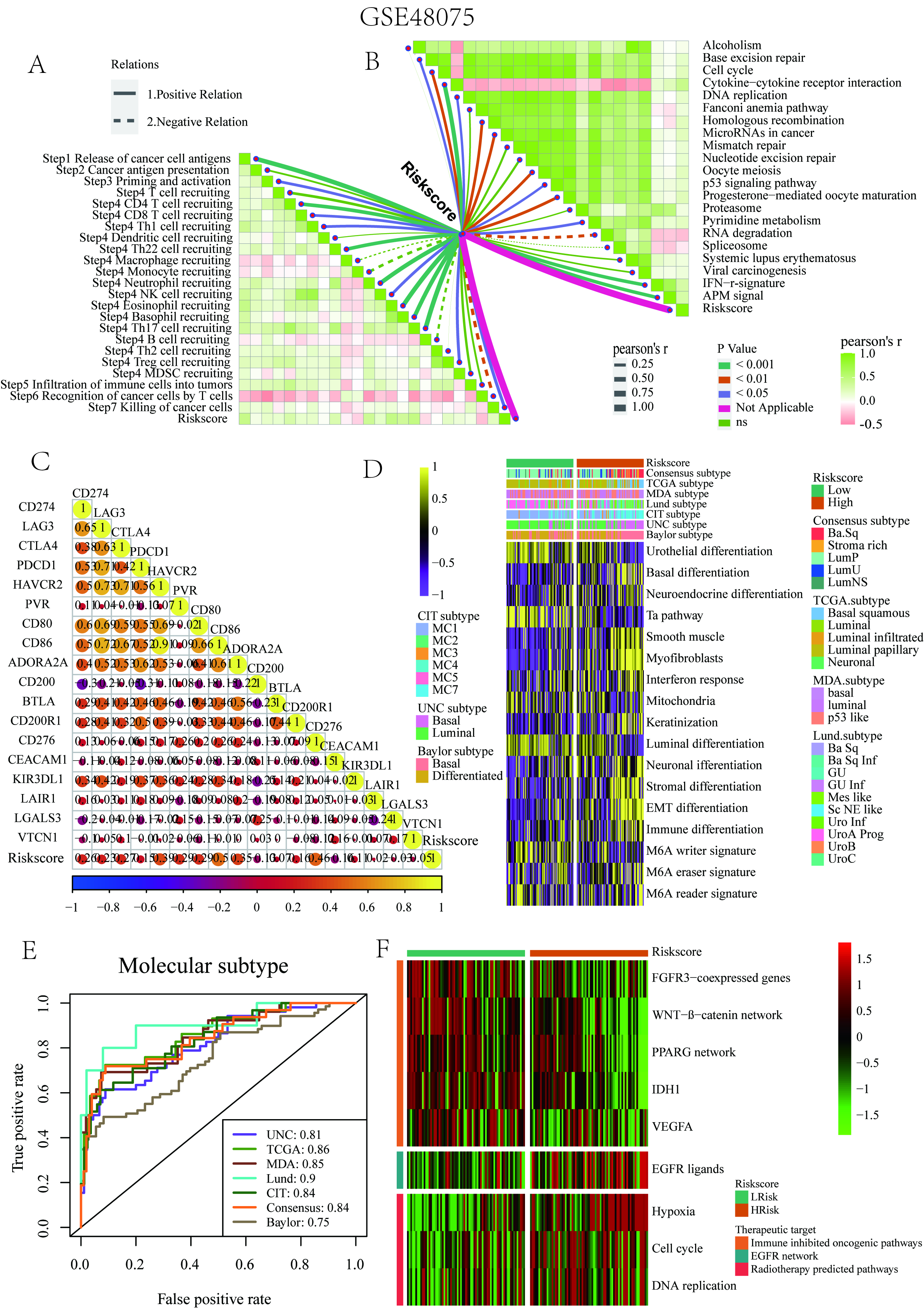

Supplement: Supplementary Figure 13 — Validation of the TGF-β risk score in the GSE48075 cohort. (A) TGF-β risk score correlated with the activities of the cancer immunity cycles. (B) The risk score correlated with the enrichment scores of ICB response related pathways. (C) The risk score correlated with the immune checkpoints. (D) The risk score accurately stratified the molecular subtypes in several different algorithms. (E) Accuracy of the risk score in predicting the molecular subtypes in several different algorithms. (F) The risk score correlated with the enrichment scores of several therapeutic signatures. [file Image_13.tif]

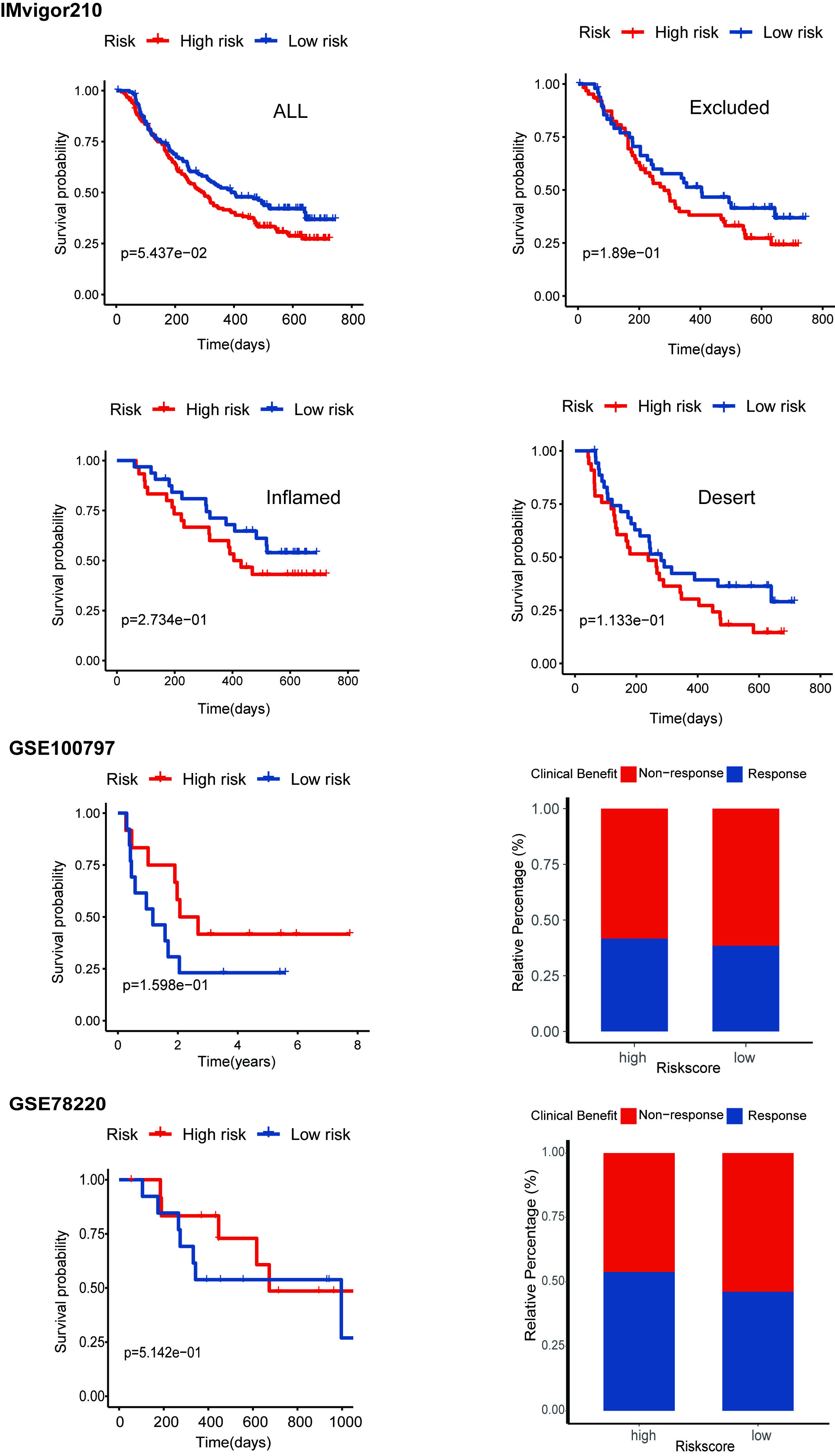

Supplement: Supplementary Figure 14 — Associations between the TGF-β risk score and therapeutic response of immunotherapy in three cohorts. [file Image_14.tif]
